# Supplementary material for: Integrin-Mediated Focal Anchorage Drives Epithelial Zippering during Mouse Neural Tube Closure
Source: Dev Cell. 2020 Feb 10;52(3):321–334.e6. doi: 10.1016/j.devcel.2020.01.012 (PMC7008250; doi:10.1016/j.devcel.2020.01.012)
Supplement: Document S1. Figures S1–S5 [file mmc1.pdf]

Developmental Cell, Volume *52*

Supplemental Information

Integrin-Mediated Focal Anchorage

Drives Epithelial Zippering

during Mouse Neural Tube Closure

Matteo A. Molè, Gabriel L. Galea, Ana Rolo, Antonia Weberling, Oleksandr Nychyk, Sandra C. De Castro, Dawn Savery, Reinhard Fässler, Patricia Ybot-González, Nicholas D.E. Greene, and Andrew J. Copp

Figure S1

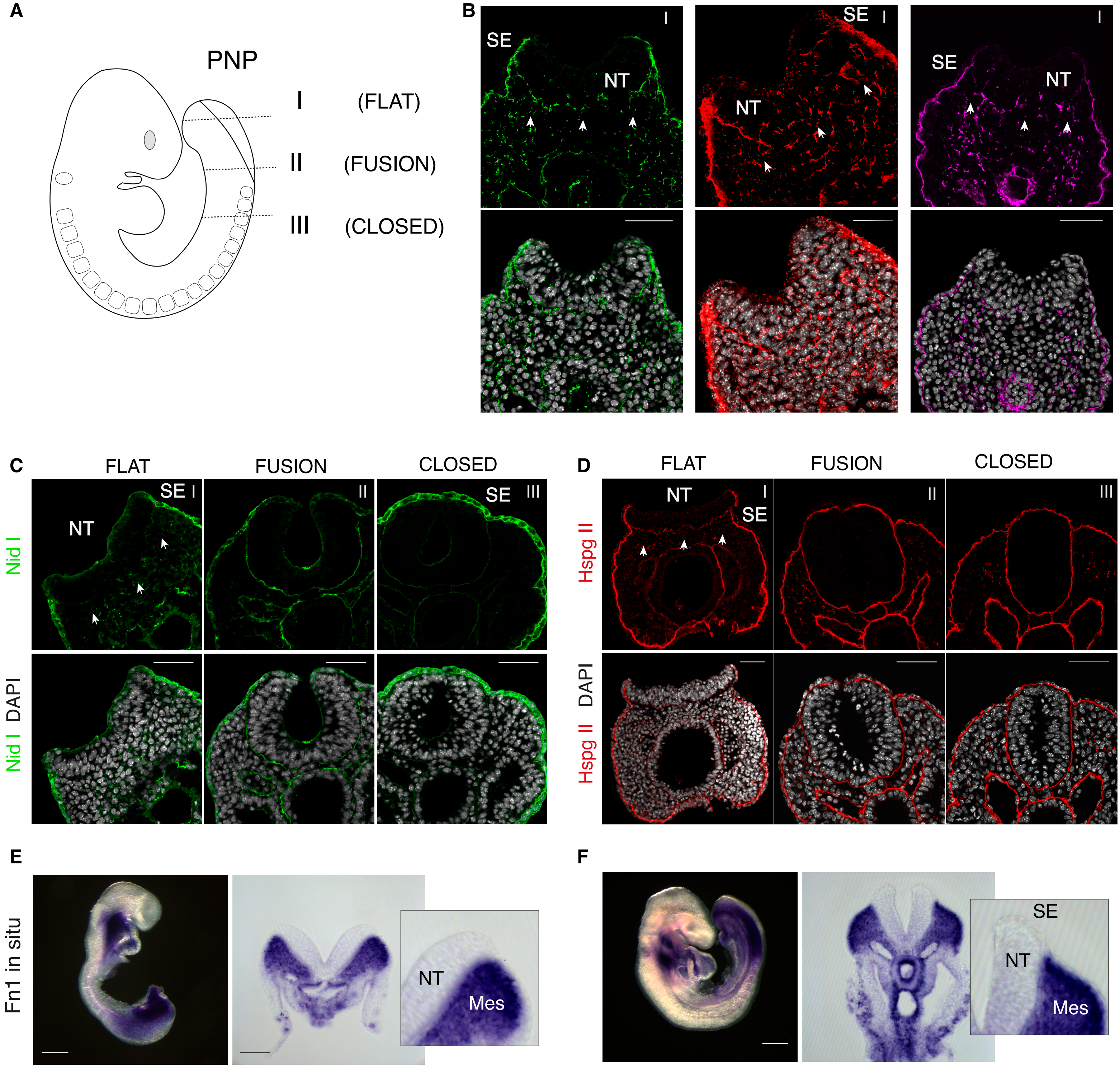
A
PNP
I
(FLAT)
II
(FUSION)
III
(CLOSED)
B
Fn1 DAPI
Lam DAPI
Col4 DAPI
SE
NT
I
C
FLAT
FUSION
CLOSED
Nid I
Nid I DAPI
II
III
D
Hspg II
Hspg II DAPI
E
Fn1 in situ
Mes
F

Figure S2

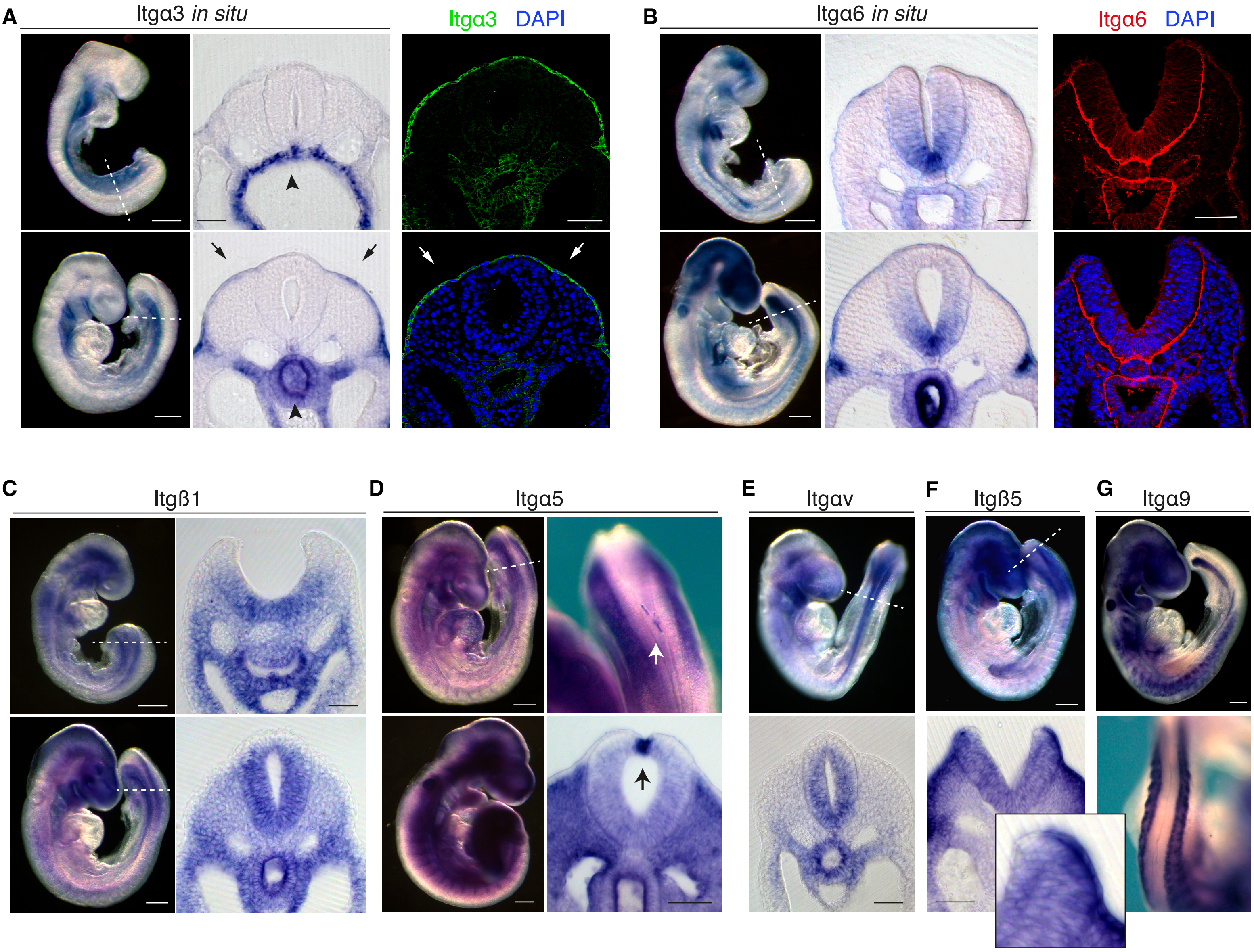

A
Itgα3 in situ
Itgα3 DAPI
B
Itgα6 in situ
Itgα6 DAPI
C
Itgβ1
D
Itgα5
E
Itgav
F
Itgβ5
G
Itgα9

Figure S3

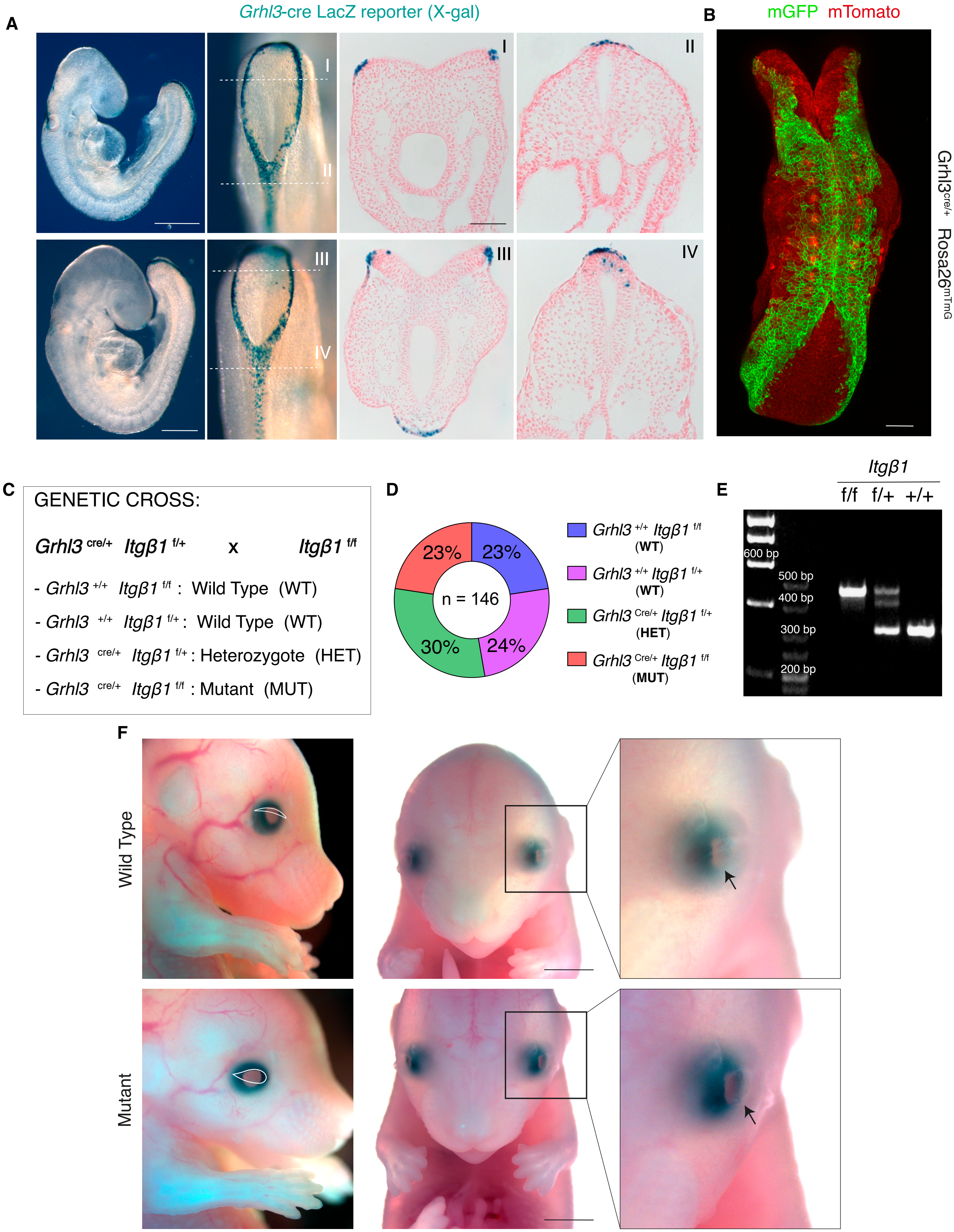

A
Grhl3-cre LacZ reporter (X-gal)
I
II
III
IV
B
mGFP mTomato
Grhl3cre/+ Rosa26mTmG
C
GENETIC CROSS:
Grhl3 cre/+ Itgβ1 f/+ x Itgβ1 f/f
- Grhl3 +/+ Itgβ1 f/f : Wild Type (WT)
- Grhl3 +/+ Itgβ1 f/+ : Wild Type (WT)
- Grhl3 cre/+ Itgβ1 f/+ : Heterozygote (HET)
- Grhl3 cre/+ Itgβ1 f/f : Mutant (MUT)
D
23%
23%
24%
30%
n = 146
Grhl3 +/+ Itgβ1 f/f (WT)
Grhl3 +/+ Itgβ1 f/+ (WT)
Grhl3 Cre/+ Itgβ1 f/+ (HET)
Grhl3 Cre/+ Itgβ1 f/f (MUT)
E
Itgβ1
f/f f/+ +/+
600 bp
500 bp
400 bp
300 bp
200 bp
F
Wild Type
Mutant

Figure S4

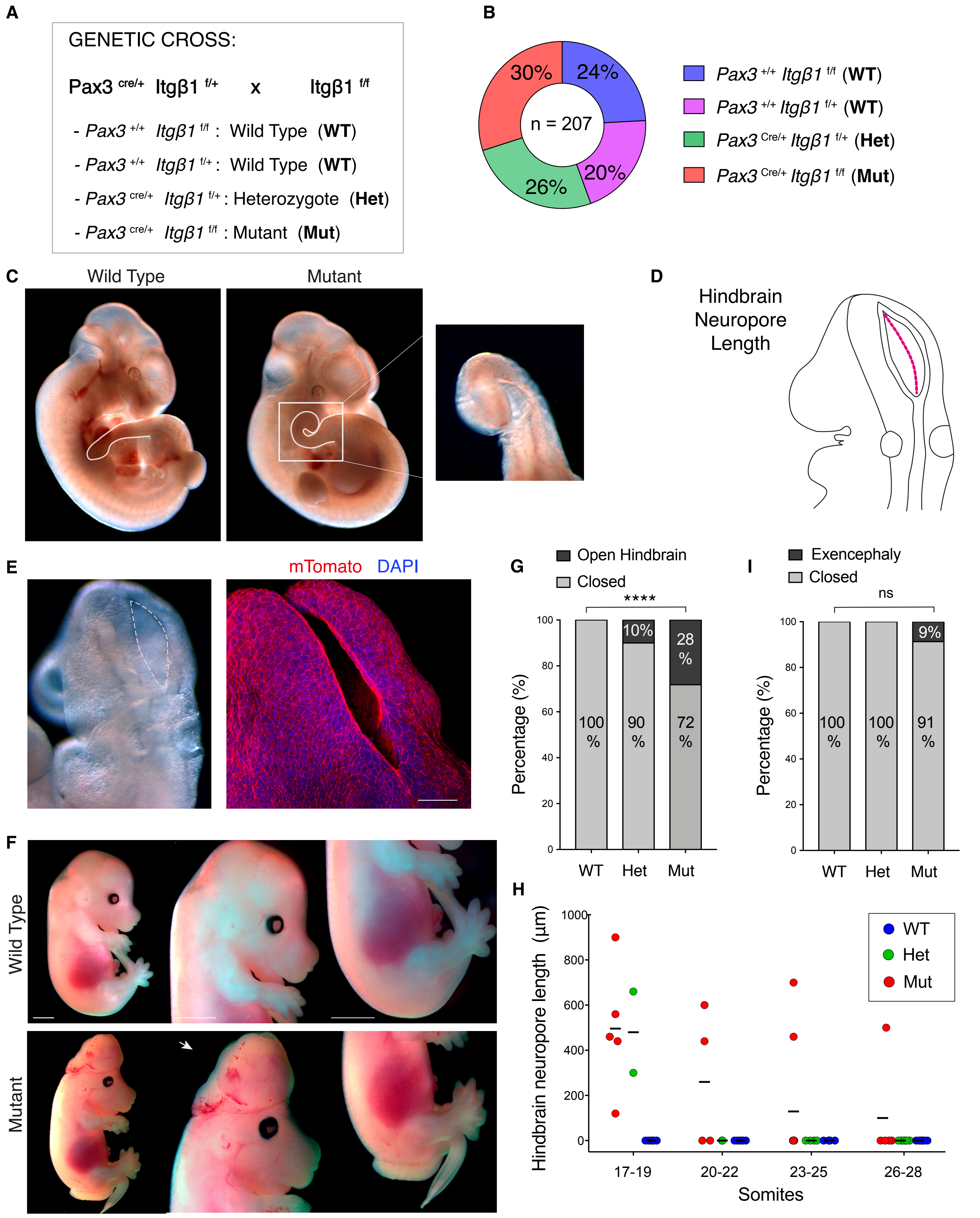
A
GENETIC CROSS:
Pax3 cre/+ Itgβ1 f/+ x Itgβ1 f/f
- Pax3 +/+ Itgβ1 f/f : Wild Type (WT)
- Pax3 +/+ Itgβ1 f/+ : Wild Type (WT)
- Pax3 cre/+ Itgβ1 f/+ : Heterozygote (Het)
- Pax3 cre/+ Itgβ1 f/f : Mutant (Mut)
B
30%
24%
n = 207
20%
26%
Pax3 +/+ Itgβ1 f/f (WT)
Pax3 +/+ Itgβ1 f/+ (WT)
Pax3 Cre/+ Itgβ1 f/+ (Het)
Pax3 Cre/+ Itgβ1 f/f (Mut)
C
Wild Type
Mutant
D
Hindbrain Neuropore Length
E
mTomato DAPI
F
Wild Type
Mutant
G
Open Hindbrain
Closed
****
Percentage (%)
100
80
60
40
20
0
100 %
10%
90 %
28 %
72 %
WT
Het
Mut
I
Exencephaly
Closed
ns
Percentage (%)
100 %
100 %
9%
91 %
WT
Het
Mut
H
Hindbrain neuropore length (µm)
1000
800
600
400
200
0
WT
Het
Mut
17-19
20-22
23-25
26-28
Somites

Figure S5

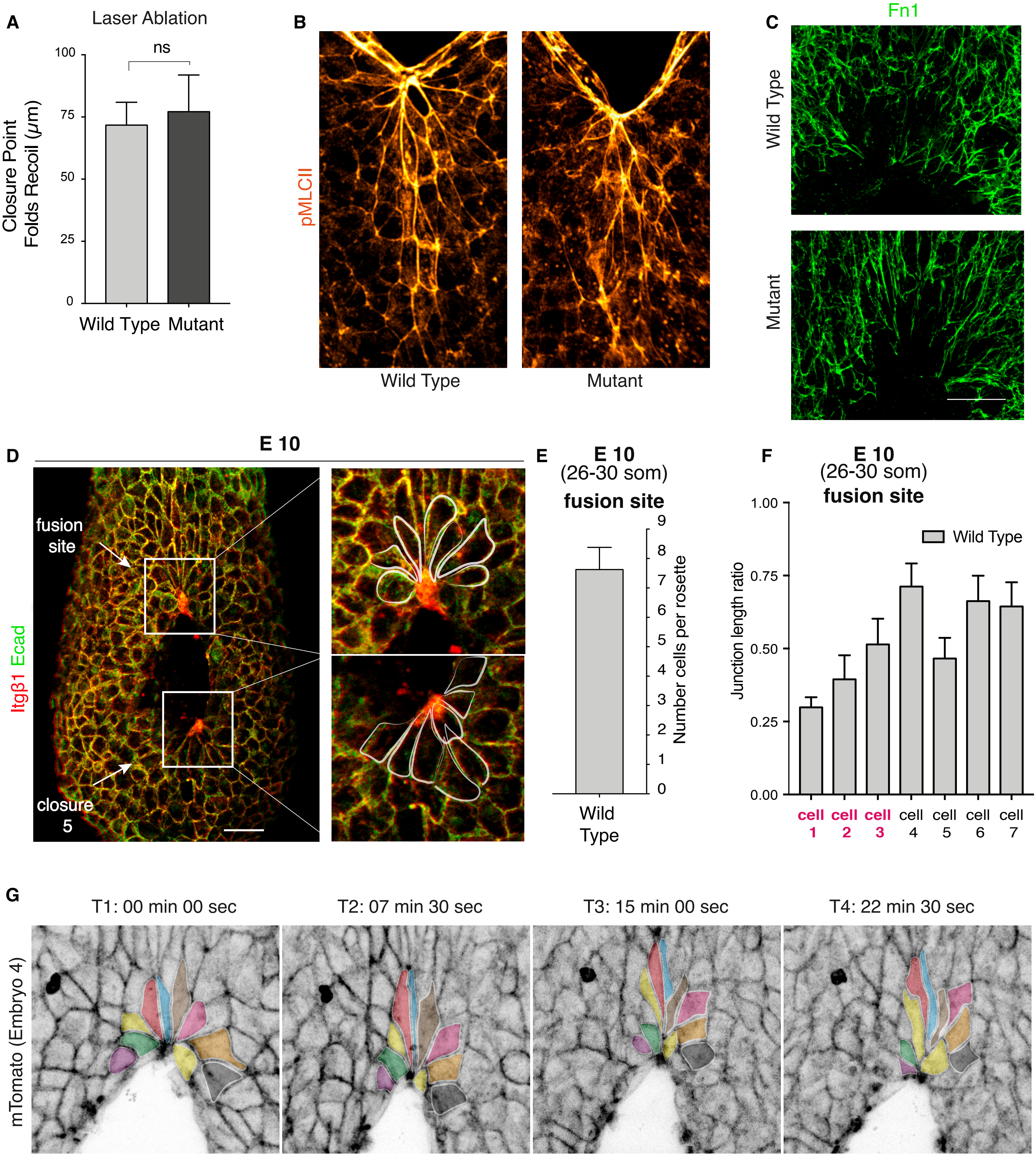

A
Laser Ablation
ns
Closure Point Folds Recoil (μm)
100
75
50
25
0
Wild Type
Mutant
B
pMLCII
Wild Type
Mutant
C
Fn1
Wild Type
Mutant
D
E 10
Itgβ1 Ecad
fusion site
closure 5
E
E 10
(26-30 som)
fusion site
Number cells per rosette
9
8
7
6
5
4
3
2
1
0
Wild Type
F
E 10
(26-30 som)
fusion site
Wild Type
Junction length ratio
1.00
0.75
0.50
0.25
0.00
cell 1
cell 2
cell 3
cell 4
cell 5
cell 6
cell 7
G
T1: 00 min 00 sec
T2: 07 min 30 sec
T3: 15 min 00 sec
T4: 22 min 30 sec
mTomato (Embryo 4)

SUPPLEMENTAL FIGURES

Figure S1. Related to Figure 1. Temporal and Spatial Heterogeneity of Basement Membranes during Neural Tube Closure. (**A**) Axial level of transverse embryonic sections: (I) 'flat' NE in the caudal PNP; (II) elevated neural folds with onset of 'fusion'; (III) recently 'closed' neural tube. (**B**) Immunofluorescence for fibronectin (Fn1), laminin (Lam) and collagen type 4 (Col4) in the most caudal PNP (I). BM deposition beneath the neuroepithelium (NT) has initiated but is fragmentary and incomplete (arrows), in contrast to the continuous BM underlying the surface ectoderm (SE) (Stages: 25 som (Fn1); 12 som (Lam); 20 som (Col4)). (**C**, **D**) Nidogen-I (Nid 1) (C) and Perlecan (Hspg II) (D) are present in the nascent BM beneath the flat NE (I, arrows), and give rise to a continuous mature BM upon elevation of the neural folds (II). Nidogen-1 expression is distributed peri-cellularly in the SE. (Stages: 21 som (C), 24 som (D)). (**E, F**) *In situ* hybridisation analysis of *Fn1*: transcripts are confined to paraxial mesoderm (Mes) and are not detectable in NE nor SE (Stages: 7 som (E); 17 som (F)). Scale bars: 50 μm (B-D); 200 μm (E-F, whole mount); 100 μm (E-F, sections).

Figure S2. Related to Figure 2. Gene Expression and Localisation of Integrins. *In situ* hybridisation (whole mount, left; sections, middle) and immunofluorescence (right) for each integrin subunit. Dotted lines on whole mounts indicate levels of sections. (**A**) *Itgα3* is transcribed in SE from 16 somite stage (arrows), and also in gut endoderm (arrowheads). Stage: 9 som (top), 16 som (bottom). Itgα3 protein shows a peri-cellular distribution around SE cells. Stage: 16 som. (**B**) *Itgα6* is transcribed in ventral neural tube (future floor plate) and SE. Stage: 9 som (top), 25 som (bottom). Itgα6 protein is intensely expressed at the basal neuroepithelial surface, with a ventro-dorsal gradient. Stage: 24 som. (**C**) *Itgβ1* is widely expressed in all tissues. Stage: 10 som (top), 19 som (bottom). See Figure 3C,D for protein expression. (**D**) *Itgα5* is transcribed in mesoderm and also at the site of neural fold fusion, as detected in whole mounts (white arrow) and sections (black arrow). Note intense expression of the Itgα5 subunit can be detected at the neural fold tips just before they come into contact, and soon after closure at the zippering point. Stage: 20 som (top), 26 som (bottom). (**E**) *Itgαv* is expressed in NE and gut endoderm. Stage: 19 som. (**F**) *Itgβ5* is transcribed in NE, mesoderm and most intensely in SE. The neural fold tips are devoid of *Itgβ5* (zoom view). Stage: 23 som. (**G**) *Itgα9* is exclusively expressed in the paraxial mesoderm. Stage: 25 som. Scale bars: 200 μm (whole mounts), 50 μm (sections).

Figure S3. Related to Figure 3. Genetic Ablation of Integrin β1 in the Surface Ectoderm by *Grhl3*-Cre. (**A**) *Grhl3*-Cre expression as detected by the endogenous LacZ cassette inserted within the *Grhl3* locus

(nuclear staining). At E9.0 (top), Cre is expressed exclusively in the dorsal SE. At E9.5 (bottom), in addition to SE, a few dorsal neuroepithelial cells are Cre-positive. Stage: 13 som (top) and 21 som (bottom). (**B**) Lineage tracing using the reporter Rosa26^{mTmG} shows *Grhl3*-Cre mediated excision (mGFP expression) in the SE of a whole-mount E9.0 embryo, whereas non-recombined cells express mTomato. Stage: 8 som. (**C**) Genetic cross used in generation of embryos lacking integrin-*β1* in the *Grhl3* expression domain. (**D**) Frequency of the four genotypes amongst all embryos (E9.5-15.5) does not differ from Mendelian expectations (Chi-square test: $p>0.05$; n = 146). (**E**) PCR genotyping of the *Itgβ1* floxed allele. (**F**) At E15.5, mutant foetuses (2/2) fail to complete eyelid closure, compared with Het (0/5) or WT (0/5), which exhibit fully closed eyelids. Scale bars: 200 μm (A, whole mount); 100 μm (A, sections, B), 2 mm (F)

Figure S4. Related to Figure 4. Genetic Ablation of Integrin β1 in the Dorsal NE by *Pax3*-Cre. (**A**) Genetic cross used in generation of embryos lacking integrin-*β1* in the *Pax3* expression domain. (**B**) Frequency of the four genotypes amongst all embryos (E9.5-14.5) does not differ from Mendelian expectation (Chi-square test: $p>0.05$; n = 207). (**C**) At E11, a proportion of Mut embryos display a small spina bifida, together with tail flexion defect, whereas WT embryos exhibit normal spine and tail development. Stage: 42 som. (**D**) Hindbrain neuropore length was measured as shown by dotted line. (**E**, **G**) At E9.5, 28% of mutant (Mut) embryos show delayed closure of the hindbrain neuropore (E), compared to WT (G; Fisher's exact test: $p<0.0001$; n-values: 52, WT; 30, Het; 39, Mut). (**H**) WT and heterozygous (Het) embryos have closed hindbrains beyond 17 and 20 somites, respectively, whereas some Mut embryos display open hindbrain at later stages. (**F**, **I**) At E14.5, 9% of mutants fail in hindbrain closure and exhibit exencephaly (F, arrow), non-significant compared with WT (I; Fisher's exact test; n-values: 40, WT; 23, Het; 23, Mut). Scale bars: 500 μm (A); 100 μm (I); 2 mm (E).

Figure S5. Related to Figure 6-7. Biomechanical, Morphometric and Live Imaging Analysis.
(**A**) Laser ablation shows identical recoil at site of closure (Mann-Whitney, $p>0.05$, no=9 WT; no=7 Mut). Stages: 20-24 som. (**B**) Analysis of phosphorylated myosin along the dorsal region of the recently closed neural tube shows no detectable change in active contractility near the site of fusion between wild type and mutant embryos. (**C**) Fibronectin fibrils in the dorsal BM orient radially towards the site of fusion and are similarly present in both WT and Mut embryos (n=11 embryos analysed). (**D**) Late in spinal closure at E10.0 (26-30 som), semi-rosette arrangements of SE cells can be visualised both at the site of rostral-to-caudal zippering and at Closure 5. Note focal expression of integrin β1 at the vertices of both semi-rosette structures. (**E**) The semi-rosette-like structure at the main site of zippering at E10.0 (26-30 som) contains on average 7 cells (number embryos: n=8 WT; Mut N/A). (**F**)

Analysis of SE junction length ratio at E10.0 (26-30 som) at the site of zippering. SE cells near the site of fusion (cells 1-3) display a progressive trend towards proximal junction shortening (wedge-shaped morphology) in contrast to cells bordering the open PNP which have a more rectangular morphology with a junction length ratio of > 0.5 (number embryos: n=8 WT (112 cells)). (**G**) Additional example of live imaging at E9.5 (embryo 4) showing proximal junction remodelling and rosette dynamics as in Figure 7H. Scale bars: 50 μm (C-D).
