# Supplementary material for: Integrin-Mediated Focal Anchorage Drives Epithelial Zippering during Mouse Neural Tube Closure
Source: Dev Cell. 2020 Feb 10;52(3):321–334.e6. doi: 10.1016/j.devcel.2020.01.012 (PMC7008250; doi:10.1016/j.devcel.2020.01.012)
Supplement: Document S2. Article plus Supplemental Information [file mmc10.pdf]

Developmental Cell

Article

Integrin-Mediated Focal Anchorage Drives Epithelial Zippering during Mouse Neural Tube Closure

Graphical Abstract

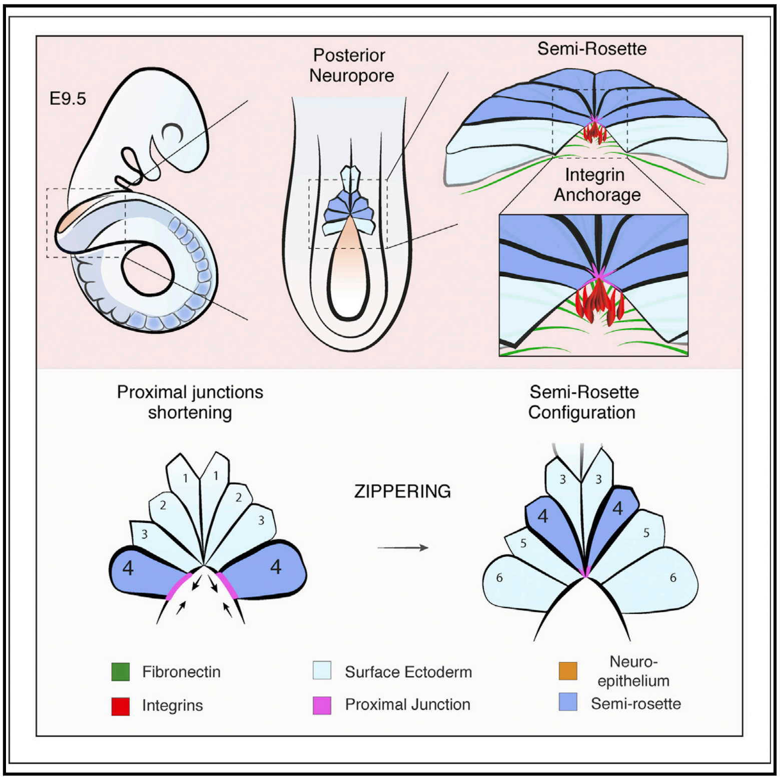

Highlights

- Surface ectoderm cells adhere at the fusion site via integrin β1 focal clustering
- Integrins mediate junction shortening and formation of a semi-rosette structure
- This configuration enables juxtaposition across the midline for fusion propagation
- Tissue-specific ablation of integrin β1 prevents zippering, causing spina bifida

Authors

Matteo A. Molè, Gabriel L. Galea, Ana Rolo, ..., Patricia Ybot-González, Nicholas D.E. Greene, Andrew J. Copp

Correspondence

m.mole.11@ucl.ac.uk (M.A.M.),
a.copp@ucl.ac.uk (A.J.C.)

In Brief

Molè et al. show that integrin-mediated basal anchorage drives epithelial zippering during mouse spinal neural tube closure. This occurs via formation of a multicellular semi-rosette configuration that promotes juxtaposition between opposing surface ectoderm junctions. The loss of integrin halts zippering progression, causing failure of neural tube closure and open spina bifida.

Molè et al., 2020, Developmental Cell 52, 321–334
February 10, 2020 © 2020 The Author(s). Published by Elsevier Inc.
https://doi.org/10.1016/j.devcel.2020.01.012

CellPress

Developmental Cell
Article

Integrin-Mediated Focal Anchorage Drives Epithelial Zippering during Mouse Neural Tube Closure

Matteo A. Molè,[1,2,*] Gabriel L. Galea,[1] Ana Rolo,[1] Antonia Weberling,[2] Oleksandr Nychyk,[1,5] Sandra C. De Castro,[1] Dawn Savery,[1] Reinhard Fässler,[3] Patricia Ybot-González,[4] Nicholas D.E. Greene,[1] and Andrew J. Copp[1,6,*]
[1]Newlife Birth Defects Research Centre, Great Ormond Street Institute of Child Health, University College London, 30 Guilford Street, London WC1N 1EH, UK
[2]Department of Physiology, Development & Neuroscience, University of Cambridge, Downing Street, Cambridge CB2 3EG, UK
[3]Department of Molecular Medicine, Max Planck Institute of Biochemistry, Am Klopferspitz 18, 82152 Martinsried, Germany
[4]Department of Neurology and Neurophysiology, Hospital Virgen de Macarena, Sevilla, Spain
[5]Neuro-endocrinology/Nutrition, Food Bioscience Department, Teagasc Moorepark, Fermoy, Co. Cork, Ireland
[6]Lead Contact
*Correspondence: m.mole.11@ucl.ac.uk (M.A.M.), a.copp@ucl.ac.uk (A.J.C.)
https://doi.org/10.1016/j.devcel.2020.01.012

SUMMARY

Epithelial fusion is a key process of morphogenesis by which tissue connectivity is established between adjacent epithelial sheets. A striking and poorly understood feature of this process is "zippering," whereby a fusion point moves directionally along an organ rudiment. Here, we uncover the molecular mechanism underlying zippering during mouse spinal neural tube closure. Fusion is initiated via local activation of integrin β1 and focal anchorage of surface ectoderm cells to a shared point of fibronectin-rich basement membrane, where the neural folds first contact each other. Surface ectoderm cells undergo proximal junction shortening, establishing a transitory semi-rosette-like structure at the zippering point that promotes juxtaposition of cells across the midline enabling fusion propagation. Tissue-specific ablation of integrin β1 abolishes the semi-rosette formation, preventing zippering and causing spina bifida. We propose integrin-mediated anchorage as an evolutionarily conserved mechanism of general relevance for zippering closure of epithelial gaps whose disturbance can produce clinically important birth defects.

INTRODUCTION

Epithelial fusion is a process of tissue morphogenesis through which pairs of epithelial sheets become apposed and eventually united at their edges to form a continuous layer. The development of numerous organs including the neural tube (NT) (Pai et al., 2012), optic fissure (Gestri et al., 2018; Patel and Sowden, 2019), palatal shelves (Greene and Pisano, 2010), tracheoesophageal foregut (Kluth and Fiegel, 2003), and presumptive genitalia (Wang and Baskin, 2008) is achieved by progression of fusion, which establishes novel tissue connectivity between apposing epithelial sheets, thereby sealing an opening. Defects in epithelial fusion typically result in the development of clinically important congenital malformations such as NT defects (NTDs), coloboma, cleft palate, tracheoesophageal fistula, and hypospadias, where the failure of fusion leaves the developing organ unsealed.

The process of epithelial fusion can be first observed during morphogenesis of the vertebrate NT, where fine coordination between elevation and fusion progression transforms the flat neural plate into a closed tube. This establishes epithelial continuity of the surface ectoderm (SE) and neuroepithelium (NE) between apposing neural folds along the entire rostro-caudal axis of the developing embryo. Primary neurulation is completed once the caudal-most region of the open NT, known as the posterior neuropore (PNP), becomes sealed. Failure to complete this last phase of spinal closure results in open spina bifida (Copp et al., 2015), a defect that arises during the first month of human embryonic development.

A particularly striking feature of epithelial fusion is the process of "zippering," in which a pair of epithelial layers becomes progressively united in one direction over a period of development. The movement of the fusion point along the organ rudiment, which is likened to the travel of a zip fastener, implies mechanical features that go beyond simply bringing together the edges of two opposing epithelia. Major insights into the cellular and molecular dynamics underlying the process of epithelial fusion originate from studies of dorsal closure in *Drosophila* and mammalian embryonic wound healing (Jacinto et al., 2001; Hayes and Solon, 2017). Two evolutionarily conserved mechanisms have been proposed (Begnaud et al., 2016). In the purse-string model, cells at the epithelial leading edge assemble a supra-cellular actomyosin cable that comes to surround the closing gap. Cable contraction results in centripetal movements of the epithelial edges, eventually sealing the gap. In the alternative cell crawling model, collective cell migration achieves gap closure as a result of lamellipodial and filopodial protrusions that emanate from the leading edges of the advancing epithelium.

In mouse NT closure, we also observed the presence of an actomyosin-containing cable that runs along the edges of the

This is an open access article under the CC BY license (http://creativecommons.org/licenses/by/4.0/).

neural folds (Galea et al., 2017). It transmits force and biomechanically couples the region of the open neural folds. However, it does not encircle the PNP until the latest stages of neurulation and is therefore unlikely to play a typical ''purse-string'' role in most of the closure. We also identified cellular protrusions, both lamellipodial and filopodial, emanating from SE cells at the zippering point of the closing PNP (Rolo et al., 2016). However, in contrast to cell crawling during dorsal closure in *Drosophila* or wound healing—where protrusions from the leading edge of the advancing epithelium crawl over an underlying tissue—protrusions during neural fold closure arise from the point of zippering and lack a substratum for crawling, as they extend into a fluid-filled gap. Hence, neither of these mechanisms appears to adequately explain the zippering process observed in mammalian neurulation.

Here, we report integrin-mediated anchorage as the cellular and molecular mechanism for fusion and zippering of the mouse NT. We show that adhesion between cells from apposing epithelia is initiated via local activation of integrin β1 and focal anchorage to a shared point of fibronectin-rich basement membrane (BM) at the zippering point, preceding the establishment of the novel cell-cell junctions. In contrast to collective migration over a substratum, focal anchorage at the site of fusion promotes local shortening of SE junctions and formation of a semi-rosette-like cellular configuration that initiates contacts between opposing cells. Loss of integrin-mediated anchorage at the fusion site prevents zippering progression, leading to the failure of NT closure and open spina bifida. Alongside the classical purse-string and cell crawling models, integrin-mediated anchorage may represent a conserved molecular mechanism employed by cells for zippering propagation and fusion of epithelial gaps *in vivo*.

RESULTS

A Fibronectin-Rich BM Forms at the Site of Neural Fold Fusion

During neurulation, the neural folds become elevated and apposed at the dorsal midline to initiate NT fusion. Concomitantly, the dorsal part of the NE exchanges its basal contact from the paraxial mesoderm (Mes) to the overlying SE (Figure 1A) (McShane et al., 2015). This event is accompanied by assembly of a novel BM at the interface between the SE and the dorsal NE (Figure 1A; elevated, magenta) (Martins-Green, 1988). We found this newly deposited BM has a distinct composition, whereas all the major structural components (Figures S1A–S1D) including collagen IV (Col4), laminins (Lam), and fibronectin (Fn1) are present around the NE (Figures 1B–1D), the novel BM forming at the very dorsal interface appears highly enriched in fibronectin fibrils (Figure 1B) while devoid of any Col4 (Figure 1C) or Lam (Figure 1D).

The strong representation of fibronectin was supported by RNA sequencing (RNA-seq) analysis, which identified *Fn1* as the most highly expressed extracellular matrix (ECM) gene at this stage of development (Figure 1E; Table S1). Even though *Fn1* is primarily transcribed within the paraxial Mes flanking the open NT (Figures S1E and S1F), it nevertheless contributes to the formation of a dense network of thick fibrils localizing precisely at the dorsal interface between the NT and the overlying SE (Figure 1B).

The unusual composition of this newly deposited BM led us to question whether localized cell-fibronectin adhesions could occur particularly at the site of neural fold fusion and whether these interactions might play a functional role during zippering of the NT.

Integrin α5β1 Is Focally Upregulated at the Site of Neural Fold Zippering

The ability of cells to interact with the BM is largely mediated by integrins: transmembrane receptors that act as primary linkage between the external ECM environment and the internal cytoskeleton (Barczyk et al., 2010; Campbell and Humphries, 2011; Lowell and Mayadas, 2012; Sun et al., 2019; Takada et al., 2007). Functional receptor complexes comprise one α and one β subunit, with 24 possible α/β combinations described in vertebrates to date.

To identify which specific integrin receptor complexes are present at the stage of NT closure, we analyzed by RNA-seq the major integrin transcripts expressed in the caudal region of mouse embryos at the 20 somite stage (Figure 2A; Table S2). *Itgβ1* (integrin β1) was the most highly expressed subunit, followed by *Itgβ5*, *Itgα5*, *Itgα3*, *Itgα6*, *Itgα9*, and *Itgαv*. This suggests that six major functional combinations are present: the complexes α5β1, αvβ1, and αvβ5, which mediate binding to RGD-containing substrates particularly fibronectin (Figure 2B, green); the receptors α6β1 and α3β1, which mediate binding to Lam isoforms (Figure 2B, red); and the receptor α9β1, which interacts primarily with vitronectin and tenascin-C. No Col4-interacting integrins were detected at this stage of development (Figure 2B, magenta).

In situ hybridization analysis (Figures 2C and S2A–S2G) revealed that the *Itgβ1* and *Itgα5* subunits were expressed significantly at the site of dorsal zippering, where the tips of the neural folds come into contact (Figure 2C, zoom-in). Importantly, integrin β1 and α5 proteins also exhibit focal clustering precisely at the site of dorsal fusion (Figure 2D). This supports a potential model where cells at this site could interact primarily with the fibronectin-rich BM through focal expression of the α5β1 integrin receptor, although potential additional interactions with other ECM ligands (Table S1) cannot be excluded (Barczyk et al., 2010; Lowell and Mayadas, 2012). A remaining question was whether such interactions involving the α5β1 receptor are mediated by the dorsal-most cells of the NE (Figure 2E, top), which might enable anchorage of the tips of the neural folds to the overlying BM, or by the dorsal-most SE cells (Figure 2E, bottom), which initiate the primary contacts between apposing neural folds at the site of fusion (Rolo et al., 2016).

Integrin β1 Adhesion at the Fusion Site Is Mediated by SE Cells and Is Necessary for NT Zippering

To investigate the tissue of origin and functional role of integrin-mediated adhesion at this site, we genetically targeted the integrin β1 subunit. Loss of this central receptor abolishes the ability of cells to interact with the dorsal fibronectin-rich BM, as β1 is the obligatory subunit for the formation of the α5β1 dimer. To overcome the early embryonic lethality of the integrin β1 knockout (Fässler and Meyer, 1995; Stephens et al., 1995), we used two different conditional approaches to confine recombination of the floxed allele of *Itgβ1* (Potocnik et al., 2000) both temporally and spatially.

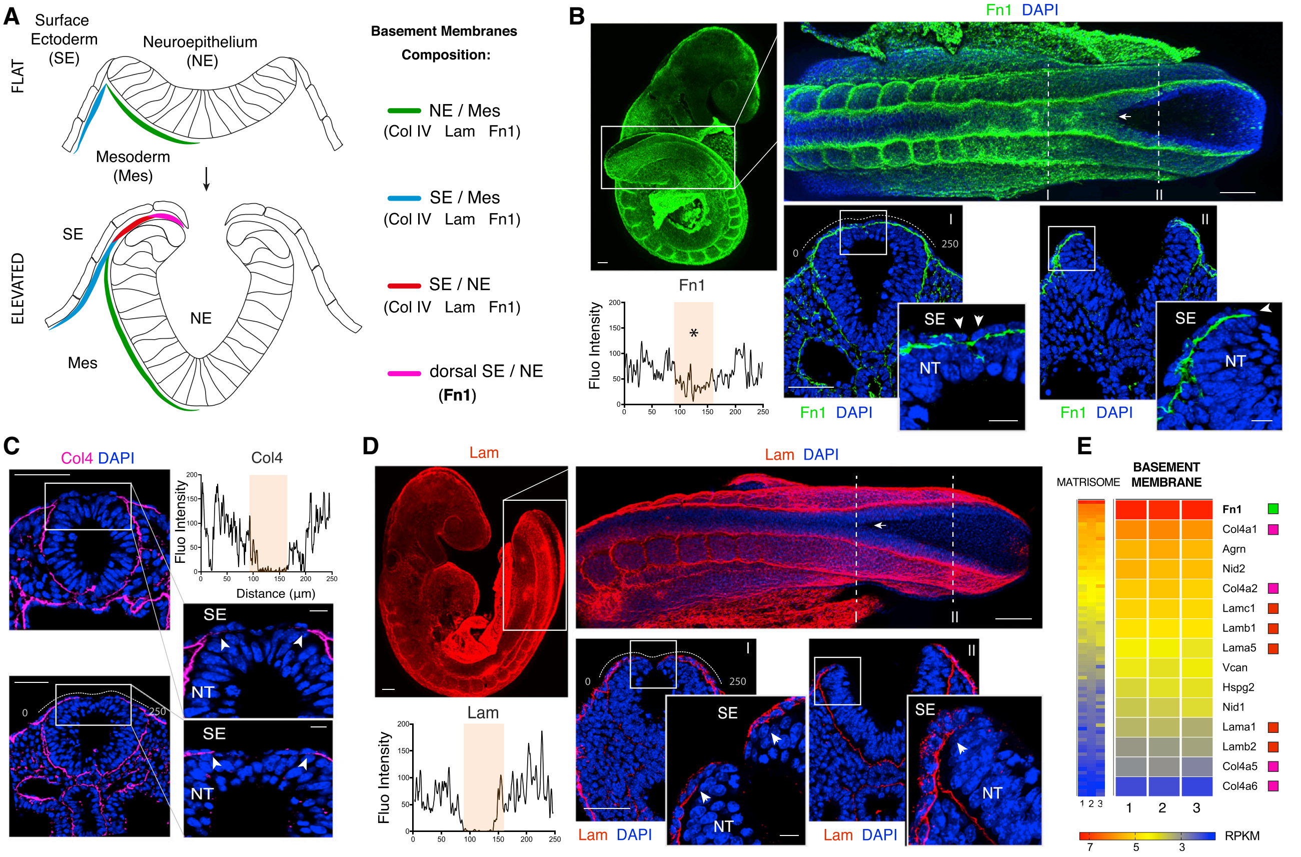

Figure 1. Molecular Composition and Distribution of Basement Membranes during Mouse Spinal NT Closure

(A) Schematic cross-sections showing basement membrane (BM) distribution. Early-forming BMs underlie NE (green) and SE (blue) during initiation of neurulation. Dorsal BMs (red and magenta) form at the interface between NE and SE as bending progresses. The dorsal-most BM (magenta) differs from other BMs in containing fibronectin but not collagen IV or laminin.

(B) Fibronectin (Fn1) forms a dense network of fibrils at the interface between SE and dorsal NE (arrow, dorsal view), in recently fused (I) and open (II) neural folds (cross-sections). Stage: 21 somites (som). Fluorescence intensity quantification (FIC) at the level of the closed NT (I) (dotted line) shows fibronectin crossing the dorsal midline (*).

(C) Collagen type IV (Col4) is not expressed in the dorsal BM (arrows indicate exclusion), as confirmed by FIC. Stages: 6 som (top) and 21 som (bottom).

(D) Laminins (Lam) are absent from the dorsal BM (arrow in the dorsal view and insets in sections), as confirmed by FIC. Stage: 18 som.

(E) RNA-seq analysis of E9.5 caudal region (20 som) for core matrisome (left: 81 out of 273 genes expressed) and a subset of BM constituents (right). *Fn1* is the most highly expressed matrisome gene. Collagen type IV is present as $\alpha1\alpha1\alpha2$, encoded by *Col4a1* and *Col4a2* genes. The major laminin trimeric combination is $\alpha5\beta1\gamma1$, encoded by *Lama5*, *Lamb1*, and *Lamc1* genes, with $\alpha1\beta1\gamma1$ also present.

Scale bars: 100 µm in (B) and (D) (whole mounts), 50 µm in (B)–(D) (sections), and 10 µm (B)–(D) (zoom).

See also Figure S1.

First, a Cre recombinase driven by the *Grhl3* promoter (Camerer et al., 2010) was used to target cells of the SE (Figures 3A, S3A, and S3B). We confirmed efficient recombination of the *Itgβ1* gene in the SE epithelium overlying the dorsal NT (Figures S3A and S3B) by activation of a promoterless *LacZ* transgene inserted at the end of the *Itgβ1* floxed locus (Figure 3B). Sporadic and scattered recombination was also observed in a few cells of the dorsal NE, as previously reported (Galea et al., 2018; Rolo et al., 2016). Nevertheless, immunofluorescence staining confirmed removal of the integrin β1 protein in the dorsal SE cells (Figure 3C), while cells of the dorsal NE were unaffected. Importantly, conditional deletion by *Grhl3-Cre* completely abolished focal expression of integrin β1 at the site of zippering (Figure 3D), supporting the hypothesis that expression of the receptor at this site is primarily mediated by cells of the SE.

At E10.5, integrin β1-deficient embryos (*Grhl3*$^{Cre/+}$; *Itgβ1*$^{f/f}$) (Figures S3C–S3E) displayed an open PNP, suggesting delayed closure, in contrast to the closed NT seen in stage-matched wild-type (WT) controls (Figure 3E). Temporal analysis of PNP length (Figure 3F) and width (Figure 3G) against developmental stage revealed a steady decrease of PNP size in WT and heterozygous embryos. In contrast, regression analysis of mutant embryos showed the rate of PNP closure to diverge significantly from controls, with cessation of PNP closure from the 20 somite stage onward.

The delay in PNP closure resulted in the development of spinal NTDs in 78% of SE-targeted integrin β1-deficient fetuses, as assessed at E14.5 (Figures 3H and 3I). The majority of mutants developed an open spina bifida phenotype associated with tail flexion defects (56%), while a minority displayed a tail flexion

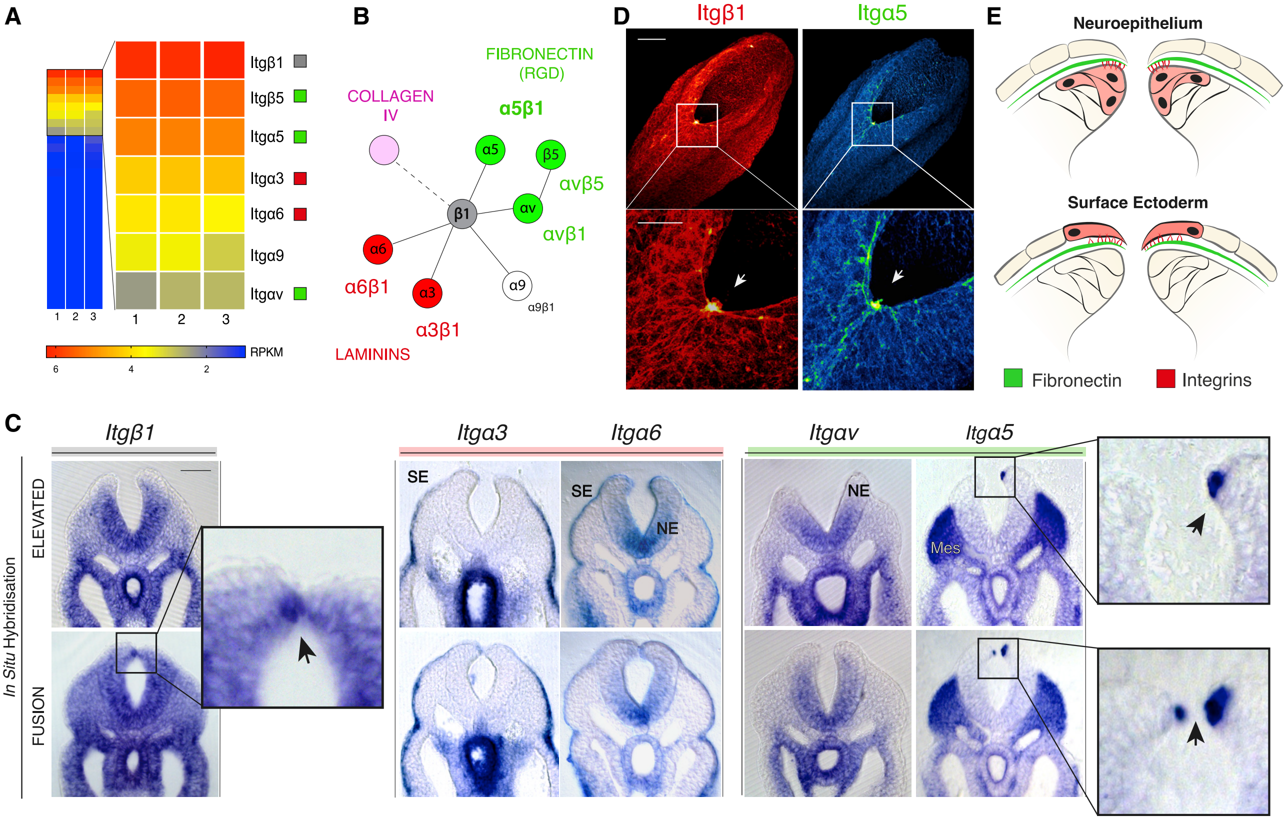

Figure 2. Integrin Expression and Localization during Spinal NT Closure

(A and B) RNA-seq analysis of integrin subunit expression in the caudal region of E9.5 (20 som) stage embryos (A). Schematic of integrin subunits expressed and functional interactions (B). Integrin β1 is the most highly expressed subunit and forms integrins α5β1 and αvβ1, which bind fibronectin (green), and integrins α3β1 and α6β1, which bind laminin (red). Subunit β5 pairs exclusively with αv to mediate binding to vitronectin, which is not expressed at this stage. The receptor α9β1 interacts with vitronectin and tenascin-C but neither are expressed. No collagen IV-interacting subunits are expressed.

(C) *In situ* hybridization analysis of integrin subunit gene expression. *Itgβ1* and *Itgα5* show an intense signal at the site of zippering where the neural fold tips come into contact (insets). *Itgαv* and *Itgα6* both show a ventro-dorsal gradient of expression in the NE, with *Itgα6* expression also in the SE. *Itgα3* is expressed in the SE only. Stages: β1 (19 som), αv (19 som), α5 (20 som), α3 (16 som), and α6 (19 som).

(D) Immunofluorescence showing expression of Itgβ1 and Itgα5 specifically at the zippering point (arrows).

(E) Diagram of neural fold tips indicating neuroepithelial (top) and SE (bottom) potential interactions of integrin α5β1 with the intervening fibronectin BM.

Scale bars: 100 μm (C); 100 μm (D); and 50 μm (D) (zoom).

See also Figure S2.

defect only (22%). The open lesion in the lumbo-sacral region of the spinal cord observed in late-stage mutant fetuses at E18 (Figure 3J) closely resembled the condition of open spina bifida (myelocele) as seen in humans. These findings demonstrate that integrin β1-mediated adhesion from cells of the SE is required for zippering and closure of the spinal NT.

Integrin β1 Upregulation at the Fusion Site Does Not Originate from Cells of the Dorsal NE

To determine whether integrin β1 expression by dorsal NE cells is also required for NT closure, we used a second conditional approach based on *Pax3*-Cre (Engleka et al., 2005) to specifically target cells of the dorsal NE (Figure 4A). X-gal staining confirmed successful recombination of the *Itgβ1* floxed allele in the dorsal NE (Figure 4B). Moreover, immunofluorescence revealed that integrin β1 expression on the basal NE surface was lost dorsally when targeted by *Pax3*-Cre (Figure 4C, between arrowheads). In contrast, integrin β1 expression in the overlying SE layer was unaffected (Figure 4C, arrows), confirming deletion in the dorsal NE region only. Importantly, in contrast to *Grhl3-Cre*-mediated deletion in the SE (Figure 3D, zoom), *Pax3*-Cre-mediated recombination in the dorsal NE failed to suppress integrin β1 focal expression (Figure 4D, zoom).

Despite the loss of NE integrin β1, mutant embryos (*Pax3*$^{Cre/+}$; *Itgβ1*$^{f/f}$) (Figures S4A and S4B) exhibited only a minor retardation of spinal NT closure, with the majority of mutant embryos showing a progressive decrease in PNP length (Figure 4E) and width (Figure 4F) with somite stage. Only 9% of mutants developed open spina bifida, while 14% displayed a mild tail flexion defect (Figures 4G and S4C). In addition to spinal neurulation, a few cases of open cranial NT (exencephaly; 9% penetrance) were observed, either alone or in combination with spinal defects (Figures S4D–S4I).

Taken together, these results suggest that the focal expression of integrin β1 at the site of fusion originates from cells of the SE rather than the NE and that integrin-mediated adhesion of SE

CellPress

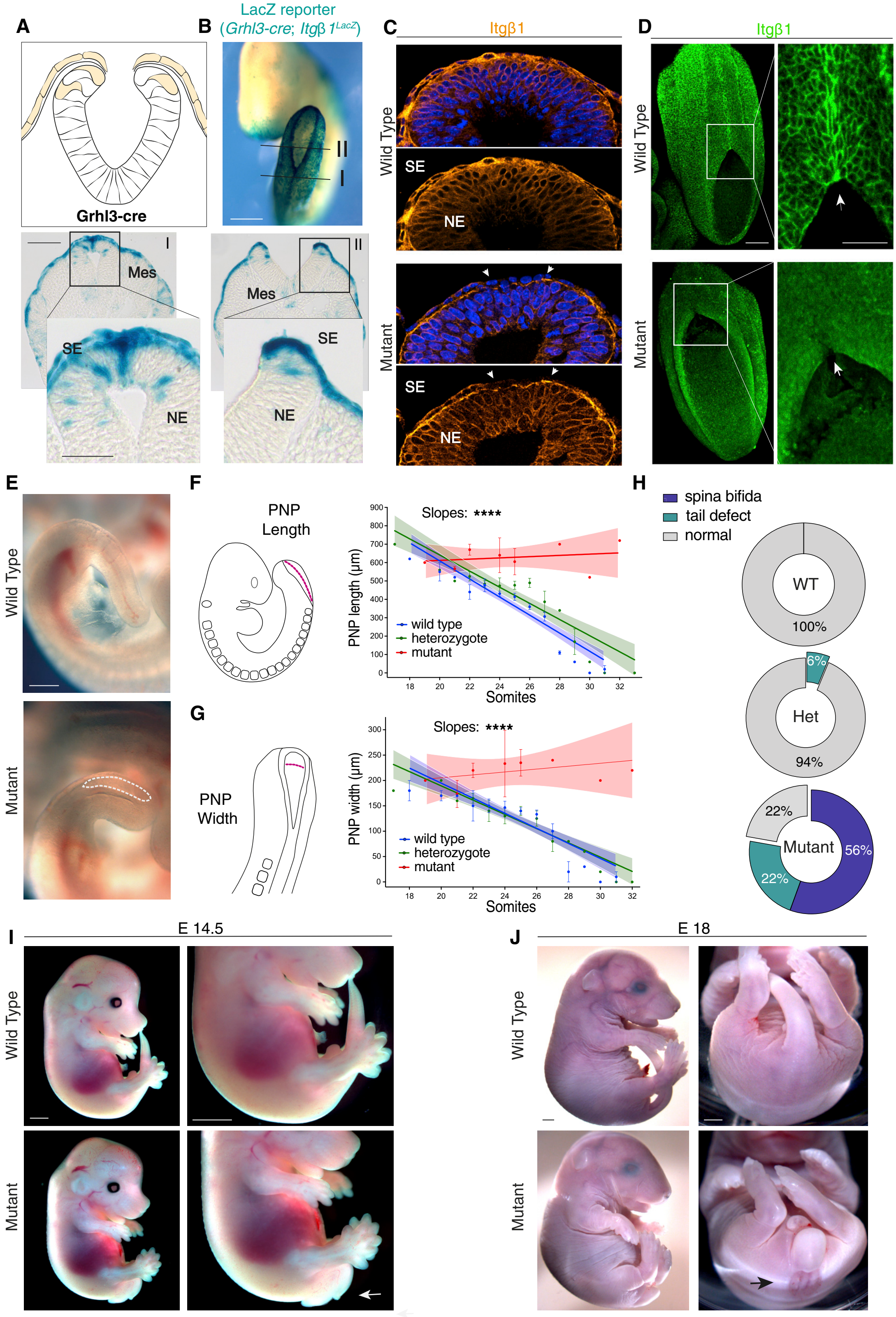

Figure 3. Genetic Ablation of Integrin β1 in SE

(A and B) *Grhl3*^{Cre}-mediated recombination of *the Itgβ1*$^{f/f}$ gene in the SE (diagram in A) as assessed by X-gal staining (B). *Grhl3*^{Cre} recombines throughout the SE and in a few dorsal NE cells. Stage: 20 som.

(legend continued on next page)

cells at this site is essential for zippering closure of the spinal NT, with highly penetrant spina bifida resulting from its absence.

Integrin β1 Is Not Required for Actomyosin Assembly, Protrusive Activity, Cell Proliferation, or Survival of SE Cells at the Fusion Site

To assess the functional role of SE-expressed integrin β1 during NT fusion, we investigated cellular events that are known to depend on integrin signaling (Barczyk et al., 2010; Campbell and Humphries, 2011; Schwartz, 2010).

Integrins have long been implicated in the regulation of actin cytoskeletal organization (Geiger et al., 2009; Hynes, 2002). Previously, we described the presence of a supra-cellular actin cable that runs along the neural fold tips, originating at the site of fusion (Galea et al., 2017). Strikingly, the cable appeared intact and normal in embryos lacking integrin β1 and both the assembly of F-actin fibers and the distribution of phosphorylated non-muscle myosin at the site of integrin focal expression appeared unchanged in mutants compared with WT controls (Figure 5A; arrow, zoom).

The site of NT fusion is also characterized by numerous protrusions from SE cells, which are essential for spinal NT zippering as their suppression leads to open spina bifida (Rolo et al., 2016). We found that deletion of integrin β1 in the SE did not disrupt protrusive activity nor did it alter the types of protrusions at the fusion site: a similar pattern of filopodia and ruffles (3-dimensional (3D) lamellipodia) was observed in both WT and mutant embryos (Figures 5B and 5C).

Integrin-mediated anchorage regulates several other critical cellular events including proliferation and survival (Harburger and Calderwood, 2009). However, neither the distribution nor frequency of SE cell divisions was affected by the loss of integrin β1 (Figures 5D and 5E). Programmed cell death is also known to be spatio-temporally associated with mammalian neurulation (Massa et al., 2009; Yamaguchi et al., 2011). However, TUNEL (terminal deoxynucleotidyl transferase dUTP nick end labeling) staining revealed only a minor increase in the number of apoptotic cells along the dorsal midline, in the region of closed NT, while a similar distribution was observed in WT and mutants at the site of fusion (Figures 5F and 5G).

This led us to conclude that integrin β1-mediated cell-matrix adhesion at the site of neural fold fusion is neither essential for actomyosin assembly and contractility nor for cell protrusive activity, proliferation, or turnover. This argues against these cellular events as potential factors leading to the failure of NT zippering in integrin-deficient embryos.

Loss of Integrin β1 Perturbs the Biomechanics and Cell Shape Properties of the Dorsal SE

In order to assess the potential effect of integrin β1 deletion on the biomechanical features of NT closure, we performed laser ablation at the site of zippering and quantified the resulting recoil due to immediate lateral displacement of the neural folds (Figures 6A and 6B). We showed previously that ablation-induced recoil is a measure of NE biomechanics, as indicated by the enhanced recoil in pre-spina bifida *Zic2* mutant embryos (Galea et al., 2017) where *Zic2* expression is restricted to the NE (Ybot-Gonzalez et al., 2007). When the zippering point of integrin β1 mutant embryos was laser ablated, we detected an almost identical degree of recoil in mutant and WT embryos, both along the entire length of the open PNP (Figure 6C) and at the site of fusion (Figure S5A). This finding argues against a potential non-cell-autonomous effect on the biomechanics of the NE, for elevation and apposition of the neural folds.

To test whether integrin β1 deficiency has a direct effect on the biomechanics of the SE epithelium, we performed a stab wound assay on the dorsal SE (Nikolopoulou et al., 2019) (Figure 6D). Mutant embryos with an SE-targeted loss of integrin β1 showed a significantly greater enlargement of the wound area than WT controls (Figure 6E), suggesting that the loss of integrin β1-mediated adhesion leads to enhanced mechanical tension within the SE epithelium.

Consistent with an increase in mechanical tension, morphometric analysis (Figure 6F) revealed a significant increase in the apical surface area of mutant SE cells (Figure 6G), even though orientation along the rostro-caudal axis was maintained (Figure 6H). The difference in size and morphology was particularly evident along the dorsal midline (Figure 6I), where a significant increase in cell width (Figure 6K) but not length (Figure 6J) appeared to have specifically contributed to the observed surface area expansion. Under normal conditions, a fine-tuned balance between cell-cell and cell-ECM adhesions must exist to safeguard the integrity of epithelia (Goodwin et al., 2016). The loss of cell-ECM adhesions may have caused an imbalance favoring cell-cell adhesion, causing enhanced biomechanical stress and cell deformation. However, these changes did not appear to result from an overall change in active contractility within the SE as shown by the comparable levels of phosphorylated non-muscle myosin in both mutant and control embryos (Figure S5B). Hence, Grhl3-Cre-mediated loss of integrin β1 function in the SE has demonstrable effects on SE biomechanics and cell shape.

(C) Immunostaining in cross-sections confirms loss of integrin β1 in the dorsal SE cells (between arrowheads).

(D) Immunostaining on whole-mount embryos confirms focal expression of integrin β1 precisely at the site of neural fold fusion in wild-type (WT) whereas integrin β1 enrichment is lost upon *Grhl3*^{Cre}-mediated recombination (mutant). Stages: 24 som, WT and 22 som, Mut.

(E) At E10.5, mutant embryos display an open PNP (dotted line), whereas the NT has closed in WT stage-matched littermates. Stages: 33 som, WT and 32 som, Mut.

(F and G) Linear regression analysis of PNP length (F) and width (G) at different somite stages in WT (n = 48; length, r = 0.79; width, r^2 = 0.60), Het (n = 37; length, r^2 = 0.69; width, r^2 = 0.66), and Mut (n = 18; length, r^2 = 0.01; width, r^2 = 0.02). Slopes of the regression lines differ significantly between WT and Het and Mut, $p < 0.0001$. Note cessation of closure in Mut from 20 somites.

(H and I) Quantification of NT defects (H) and their appearance at E14.5 (I). The majority of mutants undergo abnormal spinal NT closure, with 56% of E14.5 fetuses exhibiting open spina bifida (I, arrow) and 22% showing tail flexion defects. Fisher's exact test: $p < 0.0001$ WT versus Mut. Number of fetuses: n = 38 (WT); n = 16 (Het); and n = 18 (Mut).

(J) Open spina bifida lesions are evident perinatally (E18) in mutant fetuses at the lumbo-sacral level (arrow).

Scale bars: 500 μm (B) (whole mount); 100 μm (B) (sections, C); 50 μm (B) (sections zoom, C zoom); 500 μm (D); and 2 mm (H) and (I).

See also Figure S3.

CellPress

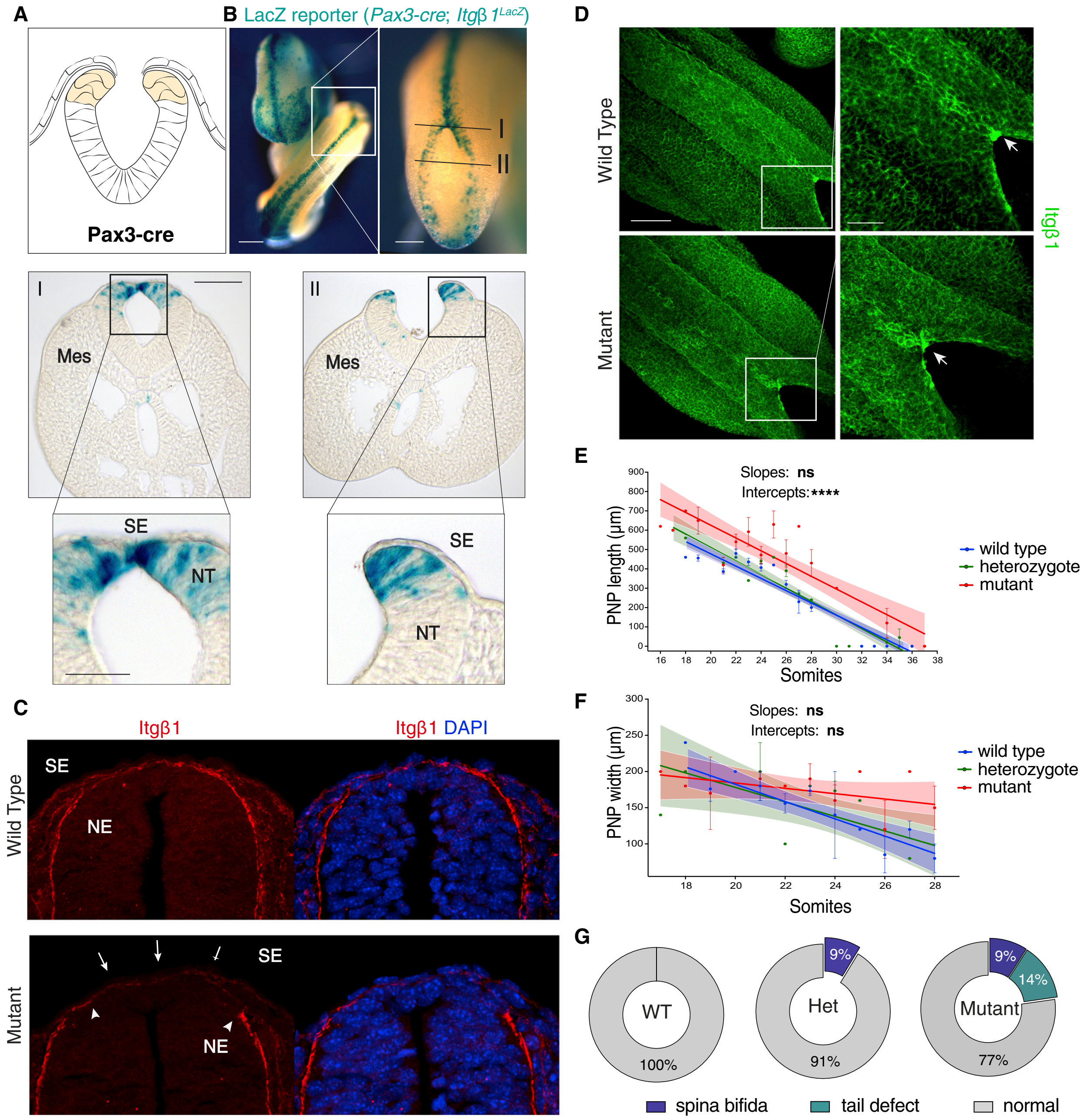

Figure 4. Genetic Ablation of Integrin β1 in the NE

(A and B) $Pax3^{Cre}$-mediated recombination of the $Itg\beta1^{f/f}$ gene in the dorsal NE (diagram in A) as assessed by whole-mount X-gal (B). $Pax3^{Cre}$ recombines *Itgβ1* in the dorsal NE along the entire body axis of the embryo in both open (II) and closed (I) NT regions. Stage: 24 som.

(C) Immunostaining in cross-sections confirms loss of integrin β1 on the basal surface of dorsal neuroepithelial cells (between arrowheads), whereas expression in the SE is unaffected (arrows).

(D) $Pax3^{Cre}$-mediated recombination of *Itgβ1* in the dorsal NE does not abolish focal upregulation of integrin β1 protein at the site of fusion.

(E and F) Linear regression analysis of PNP length (E) and width (F) by somite stage in WT (length: n = 46, r^2 = 0.89; width: n = 29, r^2 = 0.51), Het (length: n = 31, r^2 = 0.86; width: n = 15, r^2 = 0.37), and Mut embryos (length: n = 3 4, r^2 = 0.67; n = 23, r^2 = 0.09). Difference in slopes is not significant (ns). Intercept of PNP length differs significantly between WT/Het and Mut; $p < 0.0001$.

(G) At E14.5, 9% of mutant fetuses exhibit open spina bifida and 14% exhibit tail flexion defects. Fisher's exact test: p = 0.004, WT versus Mut. Open spina bifida also occurs in heterozygotes with similar frequency (9%). Number of embryos: n = 40 (WT), n = 23 (Het), and n = 22 (Mut). Stages: 22 som, WT and 23 som, Mut. Scale bars: 500 μm (B) (left); 200 μm (B) (zoom); 100 μm (B) (sections); 50 μm (B) (section zoom); 50 μm (C); 100 μm (D); and 50 μm (D) (zoom).

See also Figure S4.

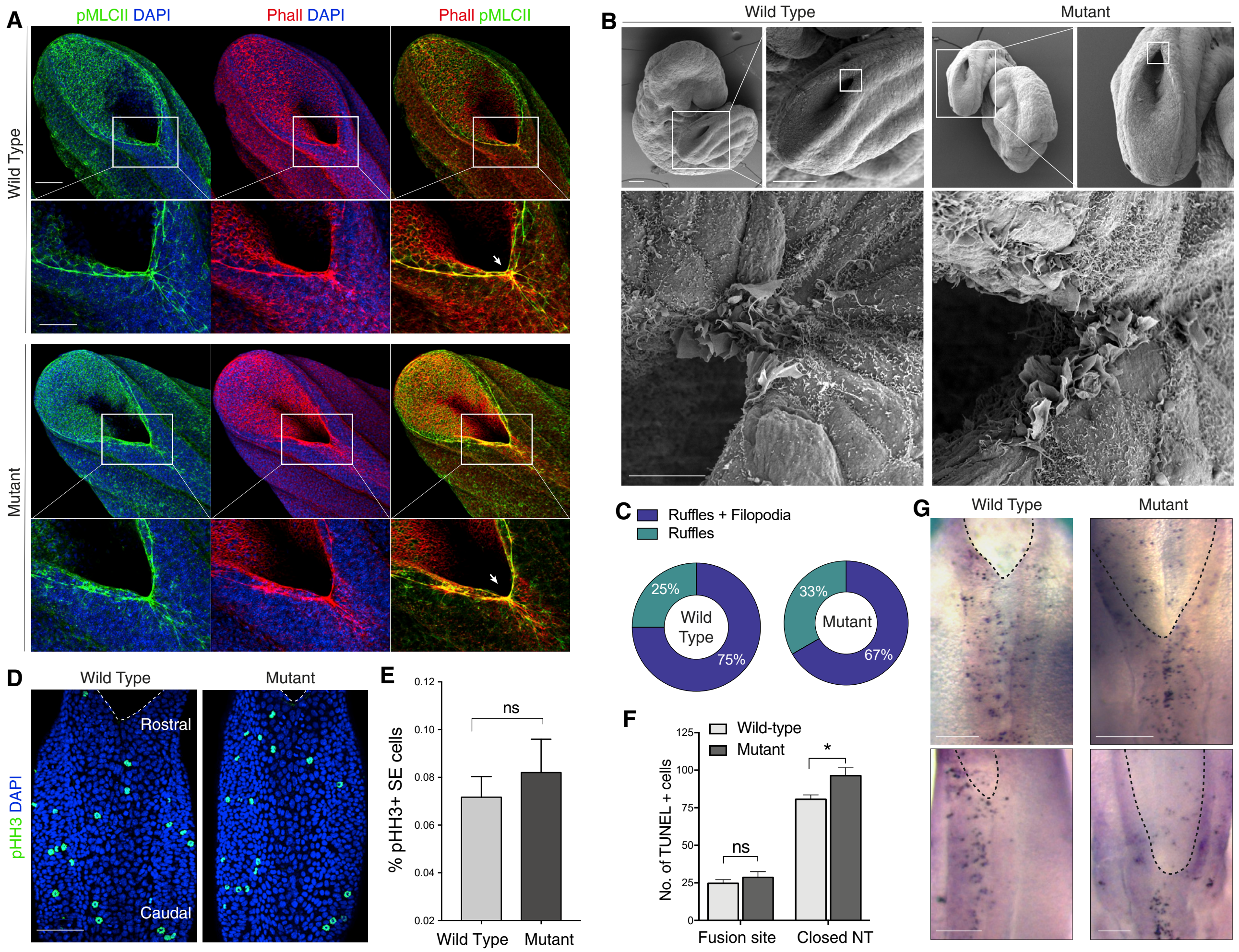

Figure 5. Cellular Analysis of Integrin β1 Deletion in the SE

Comparison of *Grhl3*^{Cre}-targeted mutants and WT embryos for cellular features previously linked to the process of spinal closure.

(A) Actomyosin cable originating at the fusion site (arrow) and running along the neural fold edges, at the SE-neuroepithelial interface. No difference is observed between WT and mutants (insets).

(B and C) Cellular protrusions (lamellipodia and filopodia) at the site of fusion, as revealed by scanning electron microscopy. Equivalent type of protrusions and distribution observed in WT and mutant embryos (Fisher's exact test, $p > 0.05$, n = 4, WT; n = 3, Mut).

(D and E) Distribution of cell divisions within the SE as detected by phospho-histone H3 staining (D) with quantification (E). WT and mutants do not differ (mitoses as % of total SE cells, Mann-Whitney Test, $p > 0.05$, n = 4, WT; n = 4, Mut).

(F and G) Programmed cell death in SE as revealed by TUNEL staining at E9.5 (G) (top) and E10.5 (G) (bottom) does not differ at the site of fusion between WT and mutants, although apoptosis is increased by ~25% over the closed NT in mutants (F: two-way ANOVA; fusion site, $p > 0.05$; closed NT, p = 0.03; n = 3, WT; n = 3, Mut). Scale bars: 100 μm (A), (B), (D), and (G, top); 50 μm (A) (zoom); 10 μm (B) (lower images); and 200 μm (G) (bottom).

Integrin-Mediated Adhesion Regulates Remodeling of SE Junctions at the Zippering Point

Insights into the cellular mechanisms of zippering morphogenesis have emerged from the analysis of NT closure in the ascidian *Ciona intestinalis* (Hashimoto et al., 2015). Sequential junctional contraction of epidermal cells ahead of the zippering point was observed to draw the next region of neural folds together, causing the zipper to move forward. Similar to *Ciona*, we found that mouse SE cells show a conserved pattern of junctional shortening as they enter the site of fusion (Figure 6L). Specifically, cells shorten their "proximal" (i.e., medial) borders, where they are attached to NE cells at the neural fold tips and adopt a characteristic wedge-shaped morphology in the dorsal view. This leads to the appearance of a highly structured semi-rosette configuration of SE cells at the zippering point (Figure 6L, zoom).

Morphometric analysis of the WT fusion site at E9.5 (19–25 somite stage) (Figures 7A–7D, WT) revealed that on average seven SE cells are in contact with the zippering point (cell numbers 1, 2, and 3 on each neural fold) and that these exhibit significantly shorter proximal junctions than cells that have not yet entered the zippering point (cell numbers 4–7) (Figures 7C and 7D). The wedge-shaped cells are arranged radially around the point of fusion, forming a semi-rosette configuration. Strikingly, the vertices of the wedge-shaped SE cells converge precisely at the focal point of integrin β1 enrichment, where cells establish basal adhesions to the underlying fibronectin-rich BM (Figure 6L;

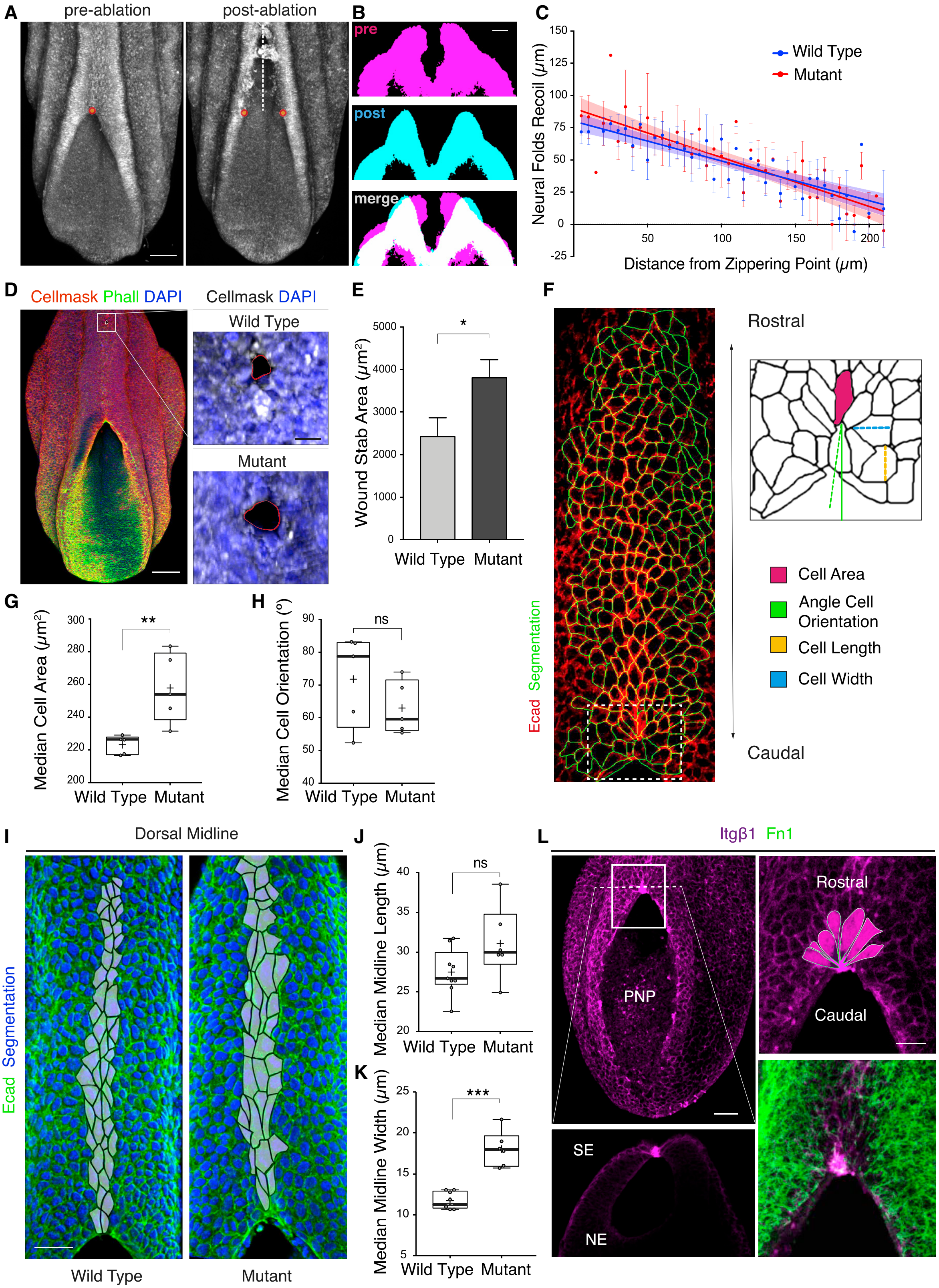

Figure 6. Biomechanical and Morphometric Analysis of Integrin β1 Deletion in SE

(A–C) Laser ablation of the zippering point (A) with imaging of the lateral recoil of the neural folds (B). WT and mutant embryos do not differ in the amount of neural fold recoil along the PNP axis (C) (linear regression: WT, $r^2 = 0.29$; Mut, $r^2 = 0.33$; difference in slopes, $p > 0.05$; n = 9, WT; n = 7, Mut). Stages: 20–24 som.

(legend continued on next page)

Video S1). This suggests that integrin-mediated anchorage at this site may mediate the process of SE junctional remodeling and semi-rosette formation.

Compared with the WT appearance, mutant embryos lacking integrin β1 do not exhibit a regular semi-rosette at the fusion site (Figure 7A, mutant). Cells in positions 1–3 maintain a significantly larger proximal-to-distal length ratio than WT (Figure 7D), consistent with the reduced number of cells that contribute to formation of the semi-rosette structure (Figure 7B). However, depletion of integrin β1 does not affect the shape of SE cells that are yet to enter the zippering point (cell numbers 5–7; Figure 7D). Similar to WT, these cells display a more "rectangular" dorsal profile.

In addition to integrin β1-depletion, we asked whether mutant embryos might also lack fibronectin at the fusion site. However, the distribution of fibronectin fibrils at the dorsal NE-SE interface appeared closely similar to the WT appearance (Figure S5C). These findings support the hypothesis that the inability of cells to establish basal adhesions at the site of zippering is due to the lack of molecular machinery required to interact with a normally formed BM rather than alterations in BM assembly.

Semi-Rosette Formation Is a General Feature of Mouse Spinal Neurulation

Next, we examined embryos at earlier and later stages than E9.5, to determine the morphology of SE cells at the zippering point as it progresses along the body axis. At E9.0 (10–18 somites), cells at the fusion point displayed a clear wedge-shaped morphology, although on average only four SE cells contributed to the semi-rosette, fewer than at E9.5 (Figures 7E–7G). Mutant SE cells exhibited an overall trend toward less shortening of proximal junctions close to the fusion site but there was no significant difference in length/width ratio compared with WT (Figure 7F). Similar number of SE cells contributed to the mutant semi-rosette at this early stage (Figure 7G).

Later, at E10.0 (26–30 somites), when spinal NT closure is almost complete, we found that SE cells adopt a prominent semi-rosette configuration similarly to E9.5, with an average of seven wedge-shaped cells around the fusion point (Figures S5D–S5F). At this stage, the enrichment of integrin β1 was evident not only at this "main" zippering point, which marks the rostral end of the closing PNP, but also at an additional site of zippering at the caudal end of the PNP (Figure S5D). This site, known as "Closure 5," likely represents an additional wave of fusion that moves in a caudal-to-rostral direction, to aid completion of spinal NT closure (Galea et al., 2017). Strikingly, SE cells also exhibited a semi-rosette configuration around the Closure 5 site, strongly suggesting that integrin-mediated basal anchorage may underlie progression of zippering at different zippering sites along the mid-lower spinal neuraxis.

Live Imaging Reveals Dynamics of SE Cells as They Transit the Fusion Point during Zippering Progression

To gain insight into the *in vivo* dynamics of zippering, we performed live imaging of SE cells at the fusion site of whole cultured E9.5 embryos (Figures 7H and S5G; Videos S2, S3, S4, S5, and S6). As previously observed in fixed embryos, cells in contact with the fusion site at the start of imaging (red and blue cells in Figure 7H; time point 1 (T1) and T2) display shorter proximal than distal junctions. Within 15 min of imaging, these cells further narrow their proximal junctions and become arranged into a semi-rosette configuration around a common vertex (Figure 7H; T3). This process of junction remodeling brings cells from the two sides of the neural folds into contact, converging at the shared site of integrin-mediated anchorage, around which the semi-rosette configuration is organized. At later time points (Figure 7H; T4 and T5), the same cells lose contact with the zippering point and become incorporated into the SE region that overlies the recently closed NT. Now, they exhibit an overall elongated shape in which both rostral and caudal ends of the cells are markedly narrowed.

Cells that were not yet in contact with the zippering point at the start of imaging (purple, yellow, and green cells in Figure 7H; T1), exhibit a broadly "rectangular" dorsal morphology, with long proximal junctions that border the open PNP. Later, these proximal junctions narrow progressively (Figure 7H; T2, T3, and T4) as they become incorporated into the semi-rosette at the fusion site.

Overall, our findings identify a sequence of cellular events that underlies zippering progression, as SE cells bordering the open PNP become sequentially incorporated into a transitory semi-rosette via integrin-mediated adhesion and junctional remodeling and then exit rostrally once closure is complete at that level.

DISCUSSION

Zippering morphogenesis, by which a fusion point propagates directionally to progressively unite a pair of epithelial sheets, is one of the most striking and yet poorly understood aspects of embryonic development. The process characterises numerous organ-forming events in mammals, and defects in zippering are likely responsible for the origin of several clinically important birth defects.

During mouse spinal neurulation, zippering is associated with an actin-containing cable that connects the unfused neural folds with the fusion point (Galea et al., 2017; Nikolopoulou et al., 2019)

(D and E) Wound stab assay, as an indicator of mechanical tension in the SE: location along body axis (D, left), typical recoil responses (D, right) and quantification (E). Mut shows a significant increase in recoil compared with WT (Mann-Whitney: $p = 0.03$; $n = 9$, WT; $n = 9$, Mut). Stage: 16–20 som.

(F–H) Morphometric analysis of the SE. Dorsal view of SE (F, left) with boxed area enlarged in diagram (F, right). Loss of integrin β1 causes a significant increase in SE cell surface area (G) while rostro-caudal orientation (H) is maintained (Mann-Whitney: median cell area, $p = 0.0079$; median cell orientation, $p > 0.05$; $n = 5$ WT embryos, 884 cells; $n = 5$ Mut embryos, $n = 1,219$ cells). Stages: 20–24 som.

(I–K) Morphometric analysis of the SE dorsal midline, with cells analysed indicated in grey (I). Loss of integrin β1 causes increased SE cell width (K) but not length (J) (Mann-Whitney: median cell width, $p = 0.0004$; median cell length, $p > 0.05$; $n = 9$ WT embryos, $n = 206$ cells; $n = 6$ Mut embryos, $n = 181$ cells).

(L) Immunostaining for integrin β1 in WT embryos at E9.5. Insets: virtual cross-section (bottom left) and dorsal views (top and bottom right). SE cells adopt a semi-rosette configuration at the zippering site, converging on the point of integrin β1 and fibronectin co-expression.

Scale bars: 100 μm (A) and (D); 50 μm (I) and (L); and 25 μm (L) (zoom).

See also Figure S5.

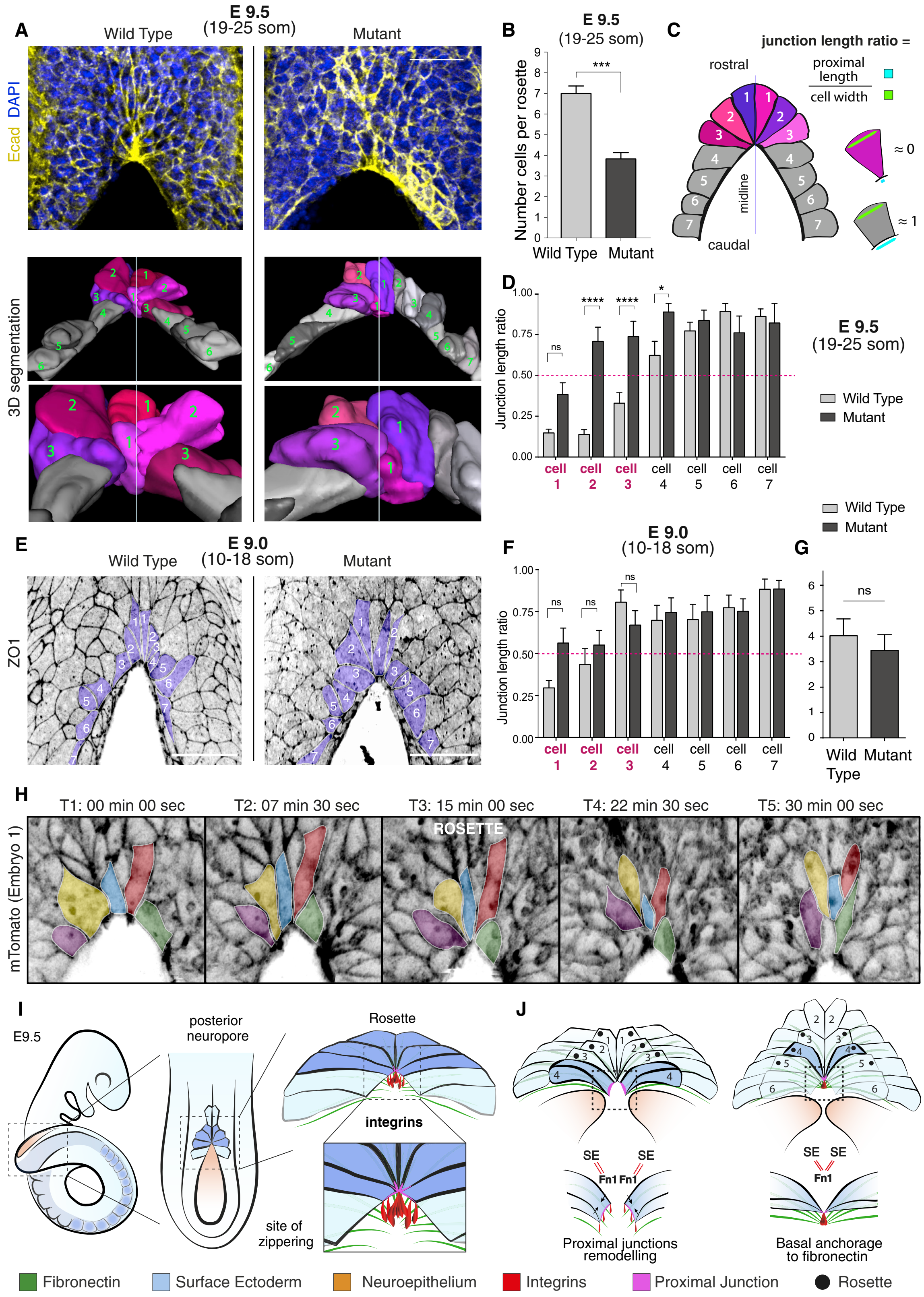

Figure 7. Cellular Arrangement at the Zippering Point in Static- and Live-Imaged Embryos

(A) At E9.5, SE cells 1–3 form a semi-rosette at the zippering point in WT embryos, as revealed by E-cadherin (Ecad) staining (top) and 3D cell reconstruction (bottom). Mutant embryos do not exhibit a regular semi-rosette.

(legend continued on next page)

and protrusions that emanate from SE cells at the zippering point (Rolo et al., 2016). These resemble similar structures implicated in fusion morphogenesis in *Drosophila* (Begnaud et al., 2016) but, while both structures participate in NT closure, neither can explain progressive zippering along the spinal axis. Here, we identify a mechanism based on integrin-mediated basal anchorage of SE cells to the BM, which mediates zippering propagation during mouse NT closure. The integrin β1 receptor exhibits focal enrichment at the contact site between apposing neural folds (Figure 7I), and coordinated adhesion toward this common site of basal anchorage drives remodeling of SE proximal junctions and the establishment of a multicellular semi-rosette-like SE structure (Figure 7I). The resulting configuration brings pairs of contralaterally positioned SE cells into close proximity (Figure 7H). This intermediate state of cell-ECM anchorage is crucial for the subsequent maturation and extension of novel cell-cell junctions between opposing cells, promoting NT closure and enabling the progression of zippering (Figure 7H).

The cellular mechanism we identify resembles that described in *Ciona intestinalis*, where sequential contraction and exchange of apical junctions bring the neural folds together, to drive the zipper forward (Hashimoto et al., 2015). However, in contrast to *Ciona*, where progressive activation of myosin II from posterior to anterior along the neural-epidermal boundary promotes rapid shortening of boundary cell junctions, contractility of the actomyosin cytoskeleton appears dispensable for mouse zippering, as both genetic and pharmacological disruption of key cytoskeletal components do not halt spinal NT closure (Escuin et al., 2015; Nikolopoulou et al., 2017).

One of the most striking findings of our study is the focal expression of integrins α5 and β1 precisely at the zippering point. Focal enrichment and activation of integrin receptors at the site of zippering has also been observed in other models of epithelial fusion. For example, during dorsal closure in *Drosophila*, high levels of the receptor βPS-integrin, the ortholog of integrin β1 in vertebrates, were detected at the advancing edge surface of the most dorsal epithelial cells (Bahri et al., 2010). Similarly, during eyelid closure in mice, integrin α5 and fibronectin were shown to be upregulated locally in the eyelid front cells as they move over the cornea (Heller et al., 2014). Most importantly, the loss of integrin-mediated adhesion in both systems leads to failure of epithelial closure (Gorfinkiel et al., 2009; Hutson et al., 2003). Further evidence comes from our study where, alongside failure of spinal NT closure, we observed incomplete eyelid closure in embryos deficient for integrin β1 (Figure S3F). This underlines the significance of integrin-mediated adhesion as a potential evolutionarily conserved mechanism in different models of epithelial zippering during embryo morphogenesis.

Overall, our findings indicate a vital role for integrin β1 in the progression of mouse spinal neurulation, with development of open spina bifida after its ablation in SE cells. This may have implications for the genetic causation of NTDs in humans, where there is known to be a strong genetic risk component (Greene and Copp, 2014; Wilde et al., 2014) and yet relatively few genes have been positively implicated in NTD aetiology (Harris and Juriloff, 2010). A recent whole-exome sequencing study of families affected by NTDs identified variants in the integrin β1-encoding gene, *ITGB1*, specifically among affected individuals, suggesting *ITGB1* as a key predisposing gene in human NTDs (Lemay et al., 2019). The strong similarity between the open lesion in our mouse model and the condition of lumbo-sacral spina bifida as observed in humans, emphasises the possibility that integrin-mediated anchorage may represent a conserved mechanism for NT zippering in humans as well as mice. Hence, integrin deficiency or impaired function could represent a potentially significant risk factor in the aetiology of open spina bifida.

STAR★METHODS

Detailed methods are provided in the online version of this paper and include the following:

- KEY RESOURCES TABLE
- LEAD CONTACT AND METERIALS AVAILABILITY
- EXPERIMENTAL MODEL AND SUBJECT DETAILS
  - Mice
- METHOD DETAILS
  - Embryo Recovery
  - Embryo Fixation
  - Embryo Genotyping
  - Whole-Mount *In Situ* Hybridisation
  - Whole-Mount TUNEL Staining
  - Immunofluorescence
  - Scanning Electron Microscopy
  - RNA-seq Analysis
  - Laser Ablation

(B) Semi-rosettes contain on average 7 cells in WT and 3–4 cells in mutants (Mann-Whitney: p = 0.0002; embryos: n = 10 WT; n = 6 Mut).
(C and D) At E9.5 (19–25 som), SE cells 1–3 display a wedge-shaped morphology (junction length ratio ≈ 0) due to extreme shortening of their proximal junctions (C)–(D) (WT). Cells bordering the open PNP (cells 4–7) exhibit a more "rectangular" morphology (junction length ratio ≈ 1; C–D [WT]). Integrin β1-deficient SE cells at the site of fusion (cells 2–3) fail to shorten proximal junctions while cells 4–7 bordering the open PNP maintain a morphology similar to WT (C)–(D) (Mut) (2-way ANOVA, post-hoc Bonferroni test; cell 1: p > 0.05; cells 2–3: p < 0.001; cell 4: p = 0.028; cells 5–7: p > 0.05; embryos: n = 10 WT (137 cells); n = 6 Mut [72 cells]).
(E–G) At E9.0 (10–18 som), WT SE cells 1–2 at the site of fusion form a smaller semi-rosette than at E9.5. Mutant SE cells 1–2 display a semi-rosette but with a less prominent wedge-shaped morphology (E). Junction length ratio does not differ from WT (F, 2-way ANOVA, post-hoc Bonferroni test; p > 0.05; embryos: n = 8 WT (112 cells); n = 7 Mut [98 cells]). Each semi-rosette contains on average 3–4 cells both in WT and mutants (G, Mann-Whitney: p > 0.05; embryos: n = 8 WT; n = 7 Mut).
(H) Live imaging SE cell dynamics during zippering. Individual cells (indicated by colors) shorten their proximal junctions over time to form the semi-rosette configuration and then exit the zippering point rostrally, with further cell elongation.
(I and J) Model of semi-rosette formation and zippering propagation. SE cells upregulate integrin α5β1 at the fusion site, with coordinated adhesion to fibronectin (SE = Fn1) causing proximal junctions to shorten forming a semi-rosette (I). Opposing junctions are brought into close proximity (J), enabling cross-midline junction formation at the site of shared basal adhesion (Se = Fn1 = SE). This propagates zippering forward with novel cell-cell junction formation (SE = SE).
Scale bars: 50 μm (A) and (E).
See also Figure S5 and Videos S2, S3, S4, S5, and S6.

- ○ X-gal Staining
- ○ Live Imaging of Zippering
- QUANTIFICATION AND STATISTICAL ANALYSIS
- DATA AND CODE AVAILABILITY

SUPPLEMENTAL INFORMATION

Supplemental Information can be found online at https://doi.org/10.1016/j.devcel.2020.01.012.

ACKNOWLEDGMENTS

We thank Paula Alexandre, Neophytos Christodoulou, Francesco Conti, Juan Pedro Martínez-Barberá, Dale Moulding, and Evanthia Nikolopoulou for valuable discussion and technical assistance. This work was supported by a PhD fellowship from the Bo Hjelt Spina Bifida Foundation, a UCL School of Life & Medical Sciences Impact PhD Studentship, and grants from the Wellcome Trust (087525 and 107474) and the Medical Research Council (J003794).

AUTHOR CONTRIBUTIONS

M.A.M. and A.J.C. conceived the study and wrote the manuscript. M.A.M., G.L.G., S.C.D.C., and A.R. performed the experiments and analyzed the data. R.F., O.N., and D.S. provided the mouse genetic expertise and resources. M.A.M. and A.W. performed the data visualization and graphics. A.J.C., N.D.E.G., and P.Y.-G. supervised the study. A.J.C. obtained funding.

DECLARATION OF INTERESTS

The authors declare no competing interests.

Received: February 2, 2019
Revised: October 24, 2019
Accepted: January 7, 2020
Published: February 10, 2020

REFERENCES

Arjonen, A., Alanko, J., Veltel, S., and Ivaska, J. (2012). Distinct Recycling of Active and Inactive β1 integrins. Traffic *13*, 610–625.

Bahri, S., Wang, S., Conder, R., Choy, J., Vlachos, S., Dong, K., Merino, C., Sigrist, S., Molnar, C., Yang, X., et al. (2010). The leading edge during dorsal closure as a model for epithelial plasticity: Pak is required for recruitment of the Scribble complex and septate junction formation. Development *137*, 2023–2032.

Barczyk, M., Carracedo, S., and Gullberg, D. (2010). Integrins. Cell Tissue Res. *339*, 269–280.

Begnaud, S., Chen, T., Delacour, D., Mège, R.M., and Ladoux, B. (2016). Mechanics of epithelial tissues during gap closure. Curr. Opin. Cell Biol. *42*, 52–62.

Camerer, E., Barker, A., Duong, D.N., Ganesan, R., Kataoka, H., Cornelissen, I., Darragh, M.R., Hussain, A., Zheng, Y.W., Srinivasan, Y., et al. (2010). Local protease signaling contributes to neural tube closure in the mouse embryo. Dev. Cell *18*, 25–38.

Campbell, I.D., and Humphries, M.J. (2011). Integrin structure, activation, and interactions. Cold Spring Harb. Perspect. Biol. *3*, 369–397.

Copp, A.J., Adzick, N.S., Chitty, L.S., Fletcher, J.M., Holmbeck, G.N., and Shaw, G.M. (2015). Spina bifida. Nat. Rev. Dis. Primers *1*, 15007.

Engleka, K.A., Gitler, A.D., Zhang, M., Zhou, D.D., High, F.A., and Epstein, J.A. (2005). Insertion of Cre into the Pax3 locus creates a new allele of Splotch and identifies unexpected Pax3 derivatives. Dev. Biol. *280*, 396–406.

Escuin, S., Vernay, B., Savery, D., Gurniak, C.B., Witke, W., Greene, N.D.E., and Copp, A.J. (2015). Rho-kinase-dependent actin turnover and actomyosin disassembly are necessary for mouse spinal neural tube closure. J. Cell Sci. *128*, 2468–2481.

Fässler, R., and Meyer, M. (1995). Consequences of lack of beta 1 integrin gene expression in mice. Genes Dev. *9*, 1896–1908.

Galea, G.L., Cho, Y.J., Galea, G., Molè, M.A., Rolo, A., Savery, D., Moulding, D., Culshaw, L.H., Nikolopoulou, E., Greene, N.D.E., and Copp, A.J. (2017). Biomechanical coupling facilitates spinal neural tube closure in mouse embryos. Proc. Natl. Acad. Sci. USA *114*, E5177–E5186.

Galea, G.L., Nychyk, O., Mole, M.A., Moulding, D., Savery, D., Nikolopoulou, E., Henderson, D.J., Greene, N.D.E., and Copp, A.J. (2018). Vangl2 disruption alters the biomechanics of late spinal neurulation leading to spina bifida in mouse embryos. Dis. Model Mech. *11*, dmm032219.

Geiger, B., Spatz, J.P., and Bershadsky, A.D. (2009). Environmental sensing through focal adhesions. Nat. Rev. Mol. Cell Biol. *10*, 21–33.

Gestri, G., Bazin-Lopez, N., Scholes, C., and Wilson, S.W. (2018). Cell behaviors during closure of the choroid fissure in the developing eye. Front. Cell. Neurosci. *12*, 42.

Goodwin, K., Ellis, S.J., Lostchuck, E., Zulueta-Coarasa, T., Fernandez-Gonzalez, R., and Tanentzapf, G. (2016). Basal cell-extracellular matrix adhesion regulates force transmission during tissue morphogenesis. Dev. Cell *39*, 611–625.

Gorfinkiel, N., Blanchard, G.B., Adams, R.J., and Martinez Arias, A. (2009). Mechanical control of global cell behaviour during dorsal closure in Drosophila. Development *136*, 1889–1898.

Greene, N.D.E., and Copp, A.J. (2014). Neural tube defects. Annu. Rev. Neurosci. *37*, 221–242.

Greene, R.M., and Pisano, M.M. (2010). Palate morphogenesis: current understanding and future directions. Birth Defects Res. C Embryo Today *90*, 133–154.

Harburger, D.S., and Calderwood, D.A. (2009). Integrin signalling at a glance. J. Cell Sci. *122*, 159–163.

Harris, M.J., and Juriloff, D.M. (2010). An update to the list of mouse mutants with neural tube closure defects and advances toward a complete genetic perspective of neural tube closure. Birth Defects Res. A Clin. Mol. Teratol. *88*, 653–669.

Hashimoto, H., Robin, F.B., Sherrard, K.M., and Munro, E.M. (2015). Sequential contraction and exchange of apical junctions drives zippering and neural tube closure in a simple chordate. Dev. Cell *32*, 241–255.

Hayes, P., and Solon, J. (2017). Drosophila dorsal closure: an orchestra of forces to zip shut the embryo. Mech. Dev. *144*, 2–10.

Heller, E., Kumar, K.V., Grill, S.W., and Fuchs, E. (2014). Forces generated by cell intercalation tow epidermal sheets in mammalian tissue morphogenesis. Dev. Cell *28*, 617–632.

Hutson, M.S., Tokutake, Y., Chang, M.S., Bloor, J.W., Venakides, S., Kiehart, D.P., and Edwards, G.S. (2003). Forces for morphogenesis investigated with laser microsurgery and quantitative modeling. Science *300*, 145–149.

Hynes, R.O. (2002). Integrins: bidirectional, allosteric signaling machines. Cell *110*, 673–687.

Jacinto, A., Martinez-Arias, A., and Martin, P. (2001). Mechanisms of epithelial fusion and repair. Nat. Cell Biol. *3*, E117–E123.

Kluth, D., and Fiegel, H. (2003). The embryology of the foregut. Semin. Pediatr. Surg. *12*, 3–9.

Lemay, P., De Marco, P., Traverso, M., Merello, E., Dionne-Laporte, A., Spiegelman, D., Henrion, É., Diallo, O., Audibert, F., Michaud, J.L., et al. (2019). Whole exome sequencing identifies novel predisposing genes in neural tube defects. Mol. Genet. Genom. Med. *7*, e00467.

Lenter, M., Uhlig, H., Hamann, A., Jenö, P., Imhof, B., and Vestweber, D. (1993). A monoclonal antibody against an activation epitope on mouse integrin chain beta 1 blocks adhesion of lymphocytes to the endothelial integrin alpha 6 beta 1. Proc. Natl. Acad. Sci. USA *90*, 9051–9055.

Lowell, C.A., and Mayadas, T.N. (2012). Overview: studying integrins in vivo. Methods Mol. Biol. *757*, 369–397.

Martins-Green, M. (1988). Origin of the dorsal surface of the neural tube by progressive delamination of epidermal ectoderm and neuroepithelium: implications for neurulation and neural tube defects. Development *103*, 687–706.

Massa, V., Savery, D., Ybot-Gonzalez, P., Ferraro, E., Rongvaux, A., Cecconi, F., Flavell, R., Greene, N.D.E., and Copp, A.J. (2009). Apoptosis is not required for mammalian neural tube closure. Proc. Natl. Acad. Sci. USA *106*, 8233–8238.

McShane, S.G., Molè, M.A., Savery, D., Greene, N.D.E., Tam, P.P.L., and Copp, A.J. (2015). Cellular basis of neuroepithelial bending during mouse spinal neural tube closure. Dev. Biol. *404*, 113–124.

Mortazavi, A., Williams, B.A., McCue, K., Schaeffer, L., and Wold, B. (2008). Mapping and quantifying mammalian transcriptomes by RNA-Seq. Nat. Methods *5*, 621–628.

Muzumdar, M.D., Tasic, B., Miyamichi, K., Li, L., and Luo, L. (2007). A global double-fluorescent Cre reporter mouse. Genesis *45*, 593–605.

Naba, A., Clauser, K.R., Ding, H., Whittaker, C.A., Carr, S.A., and Hynes, R.O. (2016). The extracellular matrix: tools and insights for the "omics" era. Matrix Biol. *49*, 10–24.

Nikolopoulou, E., Galea, G.L., Rolo, A., Greene, N.D.E., and Copp, A.J. (2017). Neural tube closure: cellular, molecular and biomechanical mechanisms. Development *144*, 552–566.

Nikolopoulou, E., Hirst, C.S., Galea, G., Venturini, C., Moulding, D., Marshall, A.R., Rolo, A., De Castro, S.C.P., Copp, A.J., and Greene, N.D.E. (2019). Spinal neural tube closure depends on regulation of surface ectoderm identity and biomechanics by Grhl2. Nat. Commun. *10*, 2487.

Pai, Y.J., Abdullah, N.L., Mohd-Zin, S.W., Mohammed, R.S., Rolo, A., Greene, N.D.E., Abdul-Aziz, N.M., and Copp, A.J. (2012). Epithelial fusion during neural tube morphogenesis. Birth Defects Res. A Clin. Mol. Teratol. *94*, 817–823.

Patel, A., and Sowden, J.C. (2019). Genes and pathways in optic fissure closure. Semin. Cell Dev. Biol. *91*, 55–65.

Potocnik, A.J., Brakebusch, C., and Fässler, R. (2000). Fetal and adult hematopoietic stem cells require beta1 integrin function for colonizing fetal liver, spleen, and bone marrow. Immunity *12*, 653–663.

Rolo, A., Savery, D., Escuin, S., de Castro, S.C., Armer, H.E.J., Munro, P.M.G., Molè, M.A., Greene, N.D.E., and Copp, A.J. (2016). Regulation of cell protrusions by small GTPases during fusion of the neural folds. eLife *5*, e13273.

Schindelin, J., Arganda-Carreras, I., Frise, E., Kaynig, V., Longair, M., Pietzsch, T., Preibisch, S., Rueden, C., Saalfeld, S., Schmid, B., et al. (2012). Fiji: an open-source platform for biological-image analysis. Nat. Methods *9*, 676–682.

Schwartz, M.A. (2010). Integrins and extracellular matrix in mechanotransduction. Cold Spring Harb. Perspect. Biol. *2*, a005066.

Stephens, L.E., Sutherland, A.E., Klimanskaya, I.V., Andrieux, A., Meneses, J., Pedersen, R.A., and Damsky, C.H. (1995). Deletion of beta 1 integrins in mice results in inner cell mass failure and peri-implantation lethality. Genes Dev. *9*, 1883–1895.

Sun, Z., Costell, M., and Fässler, R. (2019). Integrin activation by talin, kindlin and mechanical forces. Nat. Cell Biol. *21*, 25–31.

Takada, Y., Ye, X., and Simon, S. (2007). The integrins. Genome Biol. *8*, 215.

Wang, M.H., and Baskin, L.S. (2008). Endocrine disruptors, genital development, and hypospadias. J. Androl. *29*, 499–505.

Wilde, J.J., Petersen, J.R., and Niswander, L. (2014). Genetic, epigenetic, and environmental contributions to neural tube closure. Annu. Rev. Genet. *48*, 583–611.

Yamaguchi, Y., Shinotsuka, N., Nonomura, K., Takemoto, K., Kuida, K., Yosida, H., and Miura, M. (2011). Live imaging of apoptosis in a novel transgenic mouse highlights its role in neural tube closure. J. Cell Biol. *195*, 1047–1060.

Ybot-Gonzalez, P., Gaston-Massuet, C., Girdler, G., Klingensmith, J., Arkell, R., Greene, N.D.E., and Copp, A.J. (2007). Neural plate morphogenesis during mouse neurulation is regulated by antagonism of Bmp signalling. Development *134*, 3203–3211.

STAR★METHODS

KEY RESOURCES TABLE

REAGENT or RESOURCE	SOURCE	IDENTIFIER
Antibodies		
Rabbit polyclonal anti-Fibronectin	Abcam	Ab23750; RRID: AB_447655
Rabbit polyclonal anti-Laminin	Abcam	Ab11575; RRID: AB_298179
Rabbit polyclonal anti-Collagen IV	Abcam	Ab19808; RRID: AB_445160
Rat monoclonal anti-Integrin β1	Merck Millipore	MAB1997: RRID: AB_2128202
Rat monoclonal anti-Integrin β1 (active, ligand bound)	BD Biosciences	#553715: RRID: AB_395001
Rat monoclonal Anti-Integrin α5	BD Biosciences	#553319: RRID: AB_394779
Rabbit polyclonal anti-phospho Myosin Light Chain 2	Cell Signaling	#3671: RRID: AB_330248
Phalloidin 568	Thermo Fisher Scientific	A12380
Rabbit polyclonal anti-phospho Histone H3	Merck Millipore	#06-570: RRID: AB_310177
Cell Mask Green	Thermo Fisher Scientific	C37608
Mouse Monoclonal Anti-Ecadherin	BD Biosciences	#610181: RRID: AB_397580
Rabbit polyclonal anti-Nidogen I	Abcam	Ab14511; RRID: AB_301290
Rat monoclonal anti-Heparan Sulfate Proteoglycan 2	Abcam	Ab17848; RRID: AB_2119101
Rat monoclonal anti-Integrin α6	Merck Millipore	MAB1378: RRID: AB_2128317
Rabbit polyclonal anti-Integrin α3	Francesco Muntoni (UCL)	Non-commercial
Critical Commercial Assays		
TUNEL staining ApopTag TdT	Millipore	S7107
Deposited Data		
RNAsequencing data: Matrisome and BM	This paper	N/A
RNAsequencing data: Integrins	This paper	N/A
Experimental Models: Organisms/Strains		
Mouse: wild type BALB/c		N/A
Mouse: Itgb1^{tm1Ref} (Integrin β1 floxed)	(Potocnik et al., 2000)	MGI: 1926498
Mouse: Grhl3$^{tm1(cre)Cgh}$ (Grhl3-cre)	(Camerer et al., 2010)	MGI: 4430902
Mouse: Pax3$^{tm1(cre)Joe}$(Pax3-cre)	(Engleka et al., 2005)	MGI: 3573783
Mouse: Gt(ROSA)26Sor $^{tm4(ACTB-tdTomato,-EGFP)Luo}$(mTmG)	(Muzumdar et al., 2007)	MGI: 3716464
Oligonucleotides		
Itgβ1 PCR genotyping floxed vs. wild type allele: F (CTTTGCGTTGTCAGCATGGG); R (ACACTGCCATCTGCCTTTCT)	This paper	N/A
Grhl3 Cre PCR genotyping: F (GATGCAACGAGTGATGAGGTTCGC); R (ACCCTGATCCTGGCAATTTCGGC)	This paper	N/A
Pax3-cre PCR genotyping: CTGCACTCGGTGTCACG, AAGCGAGCACAGTGCGGC, GAAACAGCATTGCTGT CACTTGGTCGTGGC	This paper	N/A
In situ probe *Itgb1*: F (GCTGGGTTTCACTTTGCTGG); R (CCCATTTCCCTCATGGCACT)	This paper	N/A
In situ probe *Itga5*: F (GCTCCTCCATCTTGGCATGT); R (TAGCCGAAGTAGGAGGCCAT)	This paper	N/A
In situ probe Itgav: F (GCACGTCCTCCAGGATGTTTCT); R (TTCTGCCA CTTGGTCCGAAAT)	This paper	N/A
In situ probe Itga3: F (ACTTCCAGAAAGAGTGCGGG); R (CACTGTGCCACCAAAGAAGC)	This paper	N/A

(*Continued on next page*)

Continued

REAGENT or RESOURCE	SOURCE	IDENTIFIER
In situ probe *Itga6*: F(ATGAAAGTCTCGTGCCCGTT); R (CTCGAGAACCTGTGTTGGCT)	This paper	N/A
In situ probe *Fn1*: F (GCATCAGCCCGGATGTTAGA); R (GGTTGGTGATGAAGGGGGTC)	This paper	N/A
In situ probe *Itgb5*: F (GGACCTTTCTGCGAGTGTGA); R (TGGGCAGTTCTGTGTAGCTG)	This paper	N/A
In situ probe *Itga9*: F (ACATGGTGGTGAGCCAAGAG); R (GATCCCCACCAGCAAACTGA)	This paper	N/A
Software and Algorithms		
Imaris		http://www.bitplane.com/
Prism GraphPad 8		https://www.graphpad.com/scientific-software/prism/
Fiji (ImageJ)		https://fiji.sc/
Adobe Illustrator CC		https://www.adobe.com/uk/products/illustrator.html
StrandNGS Software		https://www.strand-ngs.com/

LEAD CONTACT AND METERIALS AVAILABILITY

Further information and requests for resources and reagents should be directed to and will be fulfilled by the Lead Contact, Andrew J. Copp (a.copp@ucl.ac.uk). This study did not generate new unique reagents.

EXPERIMENTAL MODEL AND SUBJECT DETAILS

Mice

The following mouse lines were used: inbred BALB/c line for immunofluorescence and *in situ* hybridisation analyses; integrin-β1 floxed line (Potocnik et al., 2000) was a generous gift by Reinhard Fässler and was backcrossed to the C57BL/6 background (gene symbol: ^{Itgb1tm1Ref}, MGI: 1926498); Pax3-Cre line (Engleka et al., 2005) (gene symbol: Pax3$^{tm1(Cre)Joe}$, MGI: 3573783); Grhl3-Cre line (Camerer et al., 2010) (gene symbol: Grhl3$^{tm1(Cre)Cgh}$, MGI: 4430902); reporter line mTmG (Muzumdar et al., 2007) (gene symbol: Gt(ROSA)26Sor $^{tm4(ACTB-tdTomato,-EGFP)Luo}$, MGI: 3716464). Except for BALB/c, all the above lines were maintained on the C57BL/6 background. Genetic crosses were performed as shown in Figure S3 (Ghrl3-Cre line) and Figure S4 (Pax3-Cre line): mice homozygous for the floxed *Itgβ1* allele (*Itgβ1*$^{f/f}$) were crossed with doubly heterozygous mice carrying both the Cre (either *Grhl3*-Cre or *Pax3*-Cre) and the *Itgβ1* allele ($^{Cre/+}$; *Itgβ1*$^{f/+}$). The embryonic progeny generated includes four possible genotypes: mutant ($^{Cre/+}$; *Itgβ1*$^{f/f}$), heterozygote ($^{Cre/+}$; *Itgβ1*$^{f/+}$), and two wild type genotypes ($^{+/+}$; *Itgβ1*$^{f/f}$ and $^{+/+}$; *Itgβ1*$^{f/+}$). Mouse genotypes were determined by PCR on DNA samples extracted from ear clips. Animals were kept in a pathogen-free facility at the Institute of Child Health and housed in individually ventilated cages (IVCs). Mice aged 6 to 20 weeks with an average weight of 30g were used for experiments. Mice were time-mated overnight and checked the following morning for the presence of a copulation plug, designated as embryonic day (E)0.5. All animal studies were performed according to the regulations of the UK Animals (Scientific Procedures) Act 1986 and the Medical Research Council's Responsibility in the Use of Animals for Medical Research (July 1993).

METHOD DETAILS

Embryo Recovery

Pregnant females were killed by cervical dislocation and the uterine horns were explanted at gestation stages between E8.5 and E15.5. Embryos were dissected in warmed Dulbecco's Modified Eagle's Medium (DMEM) containing 25 mM HEPES and supplemented with 10% heat inactivated Foetal Bovine Serum (FBS). Embryos were then rinsed in ice-cold phosphate buffered saline (PBS) and immersed in fixative solutions.

Embryo Fixation

Three methods were used:

1) Methanol fixation: embryos were immersed in -20°C cold DMSO:MeOH (1:5), incubated for 1h at 4°C. Samples were then stored in 100 % MeOH at -20°C.

2) Acetone fixation: embryos were immersed in -20°C cold 100% acetone, incubated for 30 min at 4°C. Samples were then stored in 100 % MeOH at -20°C.
3) PFA fixation: embryos were immersed in 4% PFA (in PBS) (pH 7.4) at 4°C, and incubated overnight at 4°C. Embryos were then dehydrated through a graded scale to 100% MeOH and stored in 100 % MeOH at -20°C.

Embryo Genotyping

DNA was extracted from yolk sac or from embryonic limb bud by proteinase K treatment as follows: 1 μl of proteinase K (10 mg/ml peqlab, 04-1071) + 24 μl of the DNA lysis buffer (peqlab Cat No 31-102-T) incubated at 55°C for 5 h, followed by inactivation at 85°C for 45 min. The extracted DNA was then used for PCR genotyping (Kit: ThermoFisher, Cat no. 18038018), by the following conditions: Integrin-β1 (Itgb1^{tm1Ref}): CTTTGCGTTGTCAGCATGGG and ACACTGCCATCTGCCTTTCT, cycles: 95°C 3 min; 35 cycles x 95°C 30 sec, 53°C 30 sec, 72°C 1 min; 72°C 5 min; band products: 500 bp (floxed allele), 300 bp (wild type allele). Pax3-Cre: CTGCACTCGGTGTCACG, AAGCGAGCACAGTGCGGC, GAAACAGCATTGCTGTCACTTGGTCGTGGC, cycles: 94°C 2 min. 32 cycles x 94°C 30 sec, 60°C 30 sec, 72°C 45 sec. 72°C 5 min; band products: 600 bp (Cre allele), 350 bp (wild type allele). Grhl3-Cre: ACCCTGATCCTGGCAATTTCGGC and GATGCAACGAGTGATGAGGTTCGC, cycles: 94°C 2 min. 30 cycles x 94°C 30 sec, 63°C 30 sec,72°C 45 sec. 72°C 5 min; band products: 500 bp (Cre allele), no band (wild type allele).

Whole-Mount *In Situ* Hybridisation

Transcript sequences (cDNA) of the genes of interest were generated by Accu-Prime Taq DNA Polymerase High Fidelity (ThermoFisher: Cat no. 12346). Fibronectin (*Fn1*) ENSMUST00000055226: forward (GCATCAGCCCGGATGTTAGA), reverse (GGTTGGTGATGAAGGGGGTC) to amplify a 498 bp product which targets all 7 splice variants of *Fn1* gene. Integrin β1 (*Itgb1*) ENSMUST00000090006: forward (GCTGGGTTTCACTTTGCTGG), reverse (CCCATTTCCCTCATGGCACT); product size: 609 bp. Integrin α5 (*Itga5*) ENSMUST00000023128, forward (GCTCCTCCATCTTGGCATGT), reverse (TAGCCGAAGTAGGAGGCCAT); product size: 535 bp. Integrin αv (*Itgav*) ENSMUST00000028499: forward (GCACGTCCTCCAGGATGTTTCT), reverse (TTCTGCCACTTGGTCCGAAAT); product size: 485 bp. Integrin β5 (*Itgb5*) ENSMUST00000115028: forward (GGACCTTTCTGCG AGTGTGA), reverse (TGGGCAGTTCTGTGTAGCTG); product size: 465 bp. Integrin α3 (*Itga3*) ENSMUST00000001548: forward (ACTTCCAGAAAGAGTGCGGG), reverse (CACTGTGCCACCAAAGAAGC); product size: 512 bp. Integrin α6 (*Itga6*) ENSMUST00000028522: forward (ATGAAAGTCTCGTGCCCGTT), reverse (CTCGAGAACCTGTGTTGGCT); product size: 542 bp. Integrin α9 (*Itga9*) ENSMUST00000044165: forward (ACATGGTGGTGAGCCAAGAG), reverse (GATCCCCACCAGCAAACTGA); product size: 440 bp. The amplified products were ligated into the pGEM-T easy vector (Promega Kit Ref A137A) and transformed in DH5α competent cells by heat shock. Single colonies were isolated in Luria Broth agar plates containing 50 μg/ml Ampicillin, 100 mM IPTG and 50 mg/ml X-Gal for white/blue screening. Plasmid DNA was extracted and linearized by restriction enzyme digestion (Promega). Digoxigenin-labelled single-stranded RNA probe was transcribed (Roche) and purified by a Chroma Spin-100 DEPC-H_2O column (Clontech). For whole mount *in situ* hybridisation: embryos were initially fixed in 4% PFA and dehydrated to 100% MeOH. Embryos were rehydrated to PBS-Tween (PBT) and bleached with 6% hydrogen peroxide in PBT for 1 h, shaking on ice. After washes in PBT, embryos were incubated in proteinase K (10 mg/mL stock) at room temperature according to the developmental stages as follows: E7.5 2.5 μg/mL (1 min), E8.5 5 μg/mL (1 min), E9.5 5 μg/mL (2 min), E10.5 5 μg/mL (7 min), E11.5 10 μg/mL (8 min). Permeabilisation reaction was stopped by glycine solution: 2 mg/mL glycine in PBT, 5 min. After washes in PBT, embryos were post-fixed in 0.2% gluteraldehyde in 4% PFA (in PBS), 20 min at room temperature, followed by washes. Hybridisation mix was prepared as follows: (50 % formamide, 5x SSC pH 4.5, 50 μg/mL yeast RNA, 1% SDS, 50 μg/mL heparin). 1 mL of pre warmed hybridisation mix (70°C) was added to each embryo and incubated at 70°C for 2 h. DIG-labelling probe was added to the hybridisation mix and embryos were incubated overnight at 70°C (hybridisation step). The following day embryos were washed 3x 30 min in solution 1 at 70°C (50% formamide, 5X SSC, 1% SDS), 2x 30 min in solution 2 at 65°C (50% formamide at 70°C, 5x SSC, 1% SDS), and finally 3x 5 min in TBST (1x TBS, 1% Tween-20, Tetramisole) at room temperature. Embryos were blocked using 10% sheep serum in TBST for 60-90 min at room temperature and incubated overnight with anti-DIG AP antibody in TBST + 1% sheep serum at 4°C. The following day embryos were washed in in TBST, followed by NTMT (100 mM NaCl, 100 mM Tris HCl pH 9.5, 50 mM $MgCl_2$, 50 mM $MgCl_2$, 1% Tween-20, Tetramisole hydrochloride) for initial equilibration. NBT (4-Nitroblue tetrazolium chloride) (4.5 μL/mL) and BCIP (5-Bromo-4-chloro-3-indoyl-phosphate) (3.5 μL/mL) were diluted in NTMT. Embryos were incubated in the above solution in dark at room temperature till the colour is fully developed. Reaction was stopped in PBT. Embryos were post-fixed in 4% PFA and imaged using a LEICA DFC490 camera on a light microscope (ZEISS Stemi SV11). Embryos were embedded in gelatin-albumin and sectioned by vibratome (Leica VT1000S) (40 μm thickness). Images of sections were acquired in Zeiss AxioCamHr brightfield microscope by differential interference contrast (DIC), Nomarski.

Whole-Mount TUNEL Staining

Embryos were initially fixed in 4% PFA and dehydrated to 100% MeOH. Embryos were rehydrated in PBT (0.1% Tween in PBS) and incubated with proteinase K solution (10 μg/ml) for permeabilisation of the tissues according to the following developmental stages: E8.5 (1 min), E9.5 (4 min), E10.5 (8 min) at room temperature. The reaction was stopped by glycine solution (2 mg/ml, 2 min) and embryos were post-fixed first in 4% PFA (20 min, room temp) followed by a mixture of ethanol and acetic acid (2:1) on ice (10 min). After incubation in equilibration buffer (ApopTag TdT enzyme kit) (1 h, room temp), embryos were incubated overnight at 37°C in working

strength TdT enzyme (80 μl TdT enzyme, 160 μl reaction buffer, 0.7 μl Triton). The reaction was stopped by incubation in stop/wash buffer (3 h). Embryos were incubated in blocking solution (5% heat-inactivated sheep serum, 2 mg/ml bovine serum albumin (BSA), in PBT) for 60 min followed by the addition of anti-digoxigenin AP-conjugated Fab fragments antibody (Roche) and incubated overnight, at 4°C. The following day, embryos were initially washed in BSA, equilibrated in NTMT (3x5 min) and incubated protected from light in developing solution (NTMT+NBT/BCIP, as previously described for in situ hybridisation) until colour develops, for few min. Reaction was stopped by PBT, followed by fixation in 4% PFA. Images were acquired using a LEICA DFC490 camera on a light microscope (ZEISS Stemi SV11).

Immunofluorescence

Whole mount immunofluorescence: after dissection, embryos were rinsed in PBS and fixed with the appropriate fixative solution. Permeabilisation of the tissues was achieved using a minimum of 0.025% Tween (for the integrin receptors) to a maximum of 0.1% Triton X-100 in PBS (PBT solution) for all the other antibodies, for 1h at room temperature with gentle agitation. Embryos were then blocked overnight in 5% BSA/PBT solution (filtered by a 0.45 μm filter prior to use) at 4°C. Blocking solution was then replaced by the primary antibody diluted at the appropriate concentration in fresh blocking solution. A volume of 150 μl solution was used for each individual embryo. Embryos were then washed 3x 1h in blocking solution, to remove excess of the primary antibodies, and incubated for 2h at room temperature in Alexa Fluor-conjugated secondary antibodies diluted 1:500 in blocking solution. Excess secondary antibody was removed by washing for 1h in blocking solution and further 2x 1h in PBT at room temperature. Finally, embryos were incubated for 1h at room temperature in DAPI diluted 0.5 μg/ml in PBT. After 2x washes in PBT, embryos were stored at 4°C in PBS with 0.1% sodium azide to prevent fungal or bacterial growth. Stained embryos were positioned and immobilised in a 2% agarose dish in PBS and imaged on a Zeiss LSM880 confocal microscope using either a 10x/NA0.5 W-Plan Apochromat dipping objective (WD 3.7 mm) or a 20x/NA1.0 Plan Apochromat dipping objective (WD 2.4 mm). Full embryos were imaged using Axiozoom technology v16 (Zeiss).

Frozen section immunofluorescence: embryos were immersed in 20% sucrose in PBS for 2h at 4°C for cryoprotection of the tissues, then incubated in 7.5% gelatine (in 20% sucrose in PBS) at 37°C for 15 min to allow penetration of the medium, and embedded in a block of gelatine after solidification. Blocks were snap frozen with -70°C isopentane and stored at -80°C until processing. Blocks were sectioned by cryostat (Leica) at 10 μm thickness and slices mounted on Superfrost Plus slides (Thermo Fisher). Removal of gelatine and rehydration of the tissues were achieved by immersing the slides in PBS for 20 min at 37°C. Slides were then covered by a volume of 200 μl per slide of 10% sheep serum, 2 % BSA (filtered) and 0.025% Tween (up to 0.1% Triton) in PBS by applying parafilm for even spreading of the solution and incubation for 1 h at room temperature inside a humidified chamber. Primary antibody was then applied after dilution in the same solution and slides incubated overnight at 4°C. The following day, excess antibody was removed by 3x washes in PBT, 5 min each. Slides were then incubated 1 h at room temperature protected by light in secondary antibody diluted 1:500. After washing, slides were incubated in DAPI diluted 0.5 μg/ml in PBT for 10 min for nuclear visualisation and finally mounted in Mowiol and sealed using a 24 x 60 mm # 1.5 coverslip.

Antibodies: Two different antibodies were used to detect the integrin β1 receptor: a rat monoclonal anti-Integrin β1 (MAB1997) that recognises the full β1 subunit and a rat monoclonal anti-Integrin β1 (BD Biosciences, 553715) that has been reported to recognise the active/ligand-bound form of the β1 subunit (Arjonen et al., 2012; Lenter et al., 1993). We did not find any difference in the pattern of staining; thus the two antibodies were used interchangeably throughout the study. A full list of antibodies used in this study can be found in the Key Resources Table.

Scanning Electron Microscopy

Embryos were fixed at 4°C in 2% glutaraldehyde, 2% PFA in 0.1 M phosphate buffer (pH7.4), and post-fixed in 1% OsO_4/1.5% $K_4Fe(CN)_6$ in 0.1 M phosphate buffer for 1.5 h. After washes in distilled water, embryos were dehydrated to 100% ethanol, followed by an acetone wash. Embryos were critical-point dried using CO_2 and mounted on aluminium stubs. After mounting, samples were coated with a layer of Au/Pd (2 nm thick) using a Gatan ion beam coater and imaged with a JEOL 7401 FEGSEM. Analysis and scoring of protrusions was carried out blind to genotype by analysis of the site of PNP fusion point at 2000x magnification, as previously reported (Rolo et al. 2016). Protrusions were categorised as: (i) ruffles (when predominantly or solely composed of membrane ruffles) or (ii) ruffles and filopodia (when a mixture of both types of protrusions was present with filopodia emanating from ruffles, or ruffles with microspikes). Presence of filopodia only, or absence of protrusions, were not observed.

RNA-seq Analysis

RNA extraction: RNA was obtained from the caudal regions of three wild type mouse embryo replicates (20 somite stage) prepared by severing the body axis at the 13-14th somite boundary. Sex of the embryos was confirmed by genotyping of *SRY* gene to ensure the presence of both sexes in the analysis: forward primer (CCGCTGCCAAATTCTTTGG), reverse primer (TGAAGCTTTTGGCTTTGAG). After washing in DEPC-PBS, the samples were snap frozen in dry ice and stored at -20°C until processed. RNA was extracted using the RNeasy Mini Kit (Qiagen Cat no 74104) and eluted in RNase-free water (Sigma). DNA contamination was removed using a DNA Removal Kit (Ambion AM1906). Quality control of RNA integrity was set at a final RIN value of 9.9-10.0 purity (out of a maximum of 10.0).

Library preparation: samples were processed using Illumina's TruSeq Stranded mRNA LT sample preparation kit (p/n RS-122-2101) according to manufacturer's instructions. Deviations from the protocol were as follows: 250 ng total RNA was used as starting

material; fragmentation was carried out for 10 min instead of 8 min; 14 cycles of PCR were used. Briefly, mRNA was isolated from total RNA using Oligo dT beads to pull down poly-adenylated transcripts. The purified mRNA was fragmented using chemical fragmentation (heat and divalent metal cation) and primed with random hexamers. Strand-specific first strand cDNA was generated using reverse transcriptase and Actinomycin D. The second cDNA strand was synthesised using dUTP instead of dTTP, to maintain strand specificity. The cDNA was then "A-tailed" at the 3' end to prevent self-ligation during the addition of the Adaptors with a complementary "T-tail". Indexing Adaptors were ligated to the A-Tailed cDNA. The adaptors contain sequences that allowed the libraries to be amplified by PCR, bind to the flow cell and be uniquely identified by way of a 6 bp index sequence. Finally a PCR was carried out to amplify only those cDNA fragments that had adaptors bound to both ends.

Sequencing: libraries to be multiplexed in the same run were pooled in equimolar quantities, calculated from qPCR and/or Bioanalyser fragment analysis. Samples were sequenced on the NextSeq 500 instrument (Illumina, San Diego, US) using a 43 bp paired end run. Samples were batched (multiplexed) in a single run, resulting in >15 million reads per sample.

Data Analysis: run data were demultiplexed and converted to fastq files using Illumina's bcl2fastq conversion software v2.16. Fastq files were aligned to the Mouse mm10 (Refseq) genome using the Tophat app in Illumina's online tool called Basespace (https://basespace.illumina.com). Raw read counts were quantified and normalised in StrandNGS Software as Reads Per Kilobase of per Million mapped reads (RPKM). This provided a means of comparing expression levels of genes between the three samples, by normalising for the length of the RNA transcripts and for the total number of reads from the sample (Mortazavi et al., 2008). A lower cut-off level of 1.5 on normalised values was applied to define the boundary of gene expression level considered to be significant. This was based on RPKM values for known caudally-expressed genes (*T* (Brachyury), *Cyp26A1, Nkx 1.2*: RPKM > 4), genes expressed rostrally at the boundary with the caudal region (*Sox1, Efna*: RPKM ~ 2) and genes expressed exclusively in the cranial region but excluded from the caudal axial level (*Tbx5, Otx1*, *Six6, Hesx1, Foxg1*: RPKM < 1). The most recent version of the matrisome (v2.0), released in 2016 (Naba et al., 2016) was used and only the core matrisome was analysed (273 genes collected).

Laser Ablation

Embryos were dissected from the amnion, positioned in wells within an agarose gel (4% agarose in DMEM), and submerged in dissection medium (10% FBS in DMEM) and maintained at 37°C throughout imaging. Fine microsurgical swaged needles (11-0 - Mersilene, TG140-6, Ethicon; 10-0 Prolene, BV75-3, Ethicon) were used to hold the embryos in place to enable a dorsal view of the open PNP. Images were captured on a Zeiss Examiner LSM880 confocal microscope using a 10x/NA0.5 W-Plan Apochromat dipping objective (WD 3.7 mm). The PNP was imaged before and after ablation by reflection using HeNe 633 (2% power), with Z-step of 4.92 μm, (speed = 8, bidirectional imaging, 1024x1024 pixels, averaging: 2). Laser ablation was performed on a Zeiss Examiner LSM880 confocal microscope using a 10x/NA0.5 W-Plan Apochromat and a MaiTai laser (800 nm wavelength, 100% laser power, 131 μs pixel dwell time, 1 iteration). A 300-500 μm long region of fused neural tube was ablated by sequential 15-20 steps of intermittent ablations from the site of fusion towards the rostral closed neural tube. Pre- and post-ablation 3D images were re-oriented and resliced by Imaris. Distance between the tips of the neural folds (PNP width) was measured in resliced z-stacks by Imaris software along 200 μl length from the fusion point along the rostro-caudal axis. Immediate recoil of neural folds was calculated as PNP width post-ablation minus PNP width pre-ablation, along the entire length of the open PNP.

X-gal Staining

After dissection, embryos were washed in PBS and fixed in freshly prepared 0.2% glutaraldehyde solution in PBS, on ice with shaking according to the embryonic stage: 30 min (E8.5), 50 min (E9.5), 1 h (E10.5), 1 h 30 min (E11.5). Embryos were then washed 3x 5 min in 0.1% Tween PBS solution, on ice. A lacZ solution was prepared as follows: 10 mM Potassium Ferrocyanide ($K_3Fe(CN)_6$, 10 mM Potassium Ferrocyanide ($K_4Fe(CN)_6$. 3 H_2O, 2 mM $MgCl_2$, 20 μl Nonidet P40 and 20 mM Tris HCl pH 7.5 in PBS. X-Gal (5-Bromo-4Chloro-3Indolyl-β-D- Galactopyranoside Sigma B4252) was dissolved in DMSO (dimethyl sulfoxide) to a concentration of 100 mg/ml of X-Gal in DMSO. This latter was eventually diluted in the lacZ solution to a final concentration of 1 mg/ml of X-Gal. LacZ solution with X-Gal was pre-warmed to 37°C to dissolve X-Gal and then passed through a 0.22 μm filter to eliminate any X-Gal precipitates. 1 ml of pre-warmed LacZ-X-Gal solution was used per embryo, with incubation on a rotating mixer at 37°C overnight, with protection from light. Embryos were post-fixed in 4% PFA and sectioned by cryostat.

Live Imaging of Zippering

Unrecombined embryos expressing membrane mTomato (Rosa26^{mTmG} reporter line (Muzumdar et al., 2007)) were immobilised on 4% agarose plates and imaged in static culture conditions by creating a small aperture through the yolk sac and amniotic membrane, thus exposing the region of open PNP as previously described (Galea et al., 2017). A microsurgical needle was placed through the allantois into the underlying agarose and a second needle under the embryo body to prevent rotation and displacement of the embryo. Culture conditions were the following: embryos were recovered in the morning of day E9.5 and incubated in rolling culture in 100% rat serum. Embryos were then transferred to 50% DMEM:50% rat serum in a humidified chamber containing 5% CO_2 in air, 37°C. Images were captured on a Zeiss Examiner LSM880 confocal microscope using a 20x/NA1 W-Plan Apochromat dipping objective (WD 3.7 mm) with the following parameters: laser power 0.8% (561 nm, DPSS 561-10), gain 650, pinhole 1.86 AU (2.8 μm section), z size= 1.42 μm (approximately 50 stacks), bidirectional imaging, 1024x1024 pixels, maximum speed, averaging: 4 (line), 8 bit. Each Z-stack acquisition was manually adjusted in x, y and z by re-centering the field of view. Each timeframe corresponds to an interval of 7 min 30 sec. Vigorous heart beating was confirmed in all embryos analysed at the end of imaging acquisition. Post-acquisition

surface subtraction (Galea et al., 2018) and segmentation were performed to visualise the SE cells. Live imaging was reproducible in multiple wild type embryos (Figures 7H and S5G; Videos S2, S3, S4, S5, and S6). We did not attempt to image mutant embryos owing to the complete lack of zippering progression at E9.5 (19-25 somites), when live-imaging proved feasible in embryo culture.

QUANTIFICATION AND STATISTICAL ANALYSIS

Statistical data analysis was performed using GraphPad Prism 7 software. Linear regression analysis was used for quantification of PNP length and width variations over somite number increase (Figures 3 and 4), and for the quantification of neural fold recoil upon laser ablation along the rostro-caudal axis of the open PNP (Figure 6). Goodness of fit for the regression model was estimated by r^2, as reported in each dataset and the p value was calculated by comparison of the slopes and intercepts of the regression models. Data presentation for linear regression was: mean ± SEM and regression model (continuous line). Comparison of the distribution of the genotypes against the expected Mendelian ratios was calculated by Chi-square test (Figures S3 and S4). Frequency of spinal NTDs (Figures 3 and 4), cranial NTDs (Figure S4) and protrusion types (Figure 5) was tested by Fisher's Exact test of wild type versus mutant embryos or non-parametric Mann-Whitney test (two-tailed) (Figures 5, 6, and 7). Comparison between multiple groups for different factors was performed by two-way ANOVA with post-hoc Bonferroni correction for multiple comparisons (Figure 7). Normality was tested by D'Agostino-Pearson omnibus test. All the measurements and analyses were performed blind to genotyping. Statistical significance: $p \leq 0.05$ was considered statistically significant (*), $p \leq 0.01$ (**), $p \leq 0.001$ (***), $p \leq 0.0001$ (****). Sample size (n) is defined as number of embryos used in each statistical analysis, unless stated otherwise (Figures 6H–6L: median of total cells is calculated per each embryo (n)). Definition of centre and dispersion: mean ± SEM (standard error of the mean) are used in all bar plots, dot plots and linear regression models. Box and whisker plots: box represent the 25th and 75th percentiles interval, line in the middle of the box the median, the cross represents the mean, whiskers showing the minimum and maximum values. Qualitative analyses of immunofluorescence and in situ hybridisation panels consider validation of the pattern of expression observed in a minimum of n=5 embryo replicates. Statistical details such p value, n number and statistical test of every experiment can be found in the corresponding figure legend.

Software. Post-acquisition processing of raw files was carried out by using Fiji software (Schindelin et al., 2012) for brightness adjustments, cropping, outlier removal, quantifications, drift correction. Quantification of fluorescence staining in sections was performed on confocal Z slices using Fiji: mean grey values were quantified along the basal perimeter between the dorsal neural tube and overlying surface ectoderm. 3D volume rendering and re-slicing (laser ablation quantification) was performed on Imaris software. 3D reconstruction in Figure 7 was carried out manually: cells were segmented using the Fiji software, hyperstacks were assembled to 3D images through the 3DSlicer software. No quantification in 3D segmented cells was performed. Our previously-reported Surface Subtraction macro (Galea et al., 2018, available at goo.gl/zcpZkH) was used to digitally dissect the surface ectoderm E-cadherin staining from underlying background signal. The ImageJ Tissue Analyser semi-automated cell border segmentation plugin (PMID 27730585) was then used to quantify cell dimensions and orientation. Morphometric analysis was carried out in 2D on max projected and surface subtracted images and does not take into account 3D segmentation. Adobe Illustrator was used for panel assembly and schematics.

DATA AND CODE AVAILABILITY

The published article includes all RNA-seq datasets generated and analyzed during this study. Original data for Figure 1 (matrisome RNA-seq datasets) and Figure 2 (integrin subunits RNA-seq datasets) in the paper is available in Tables S1 and S2.

Developmental Cell, Volume *52*

Supplemental Information

Integrin-Mediated Focal Anchorage

Drives Epithelial Zippering

during Mouse Neural Tube Closure

Matteo A. Molè, Gabriel L. Galea, Ana Rolo, Antonia Weberling, Oleksandr Nychyk, Sandra C. De Castro, Dawn Savery, Reinhard Fässler, Patricia Ybot-González, Nicholas D.E. Greene, and Andrew J. Copp

Figure S1

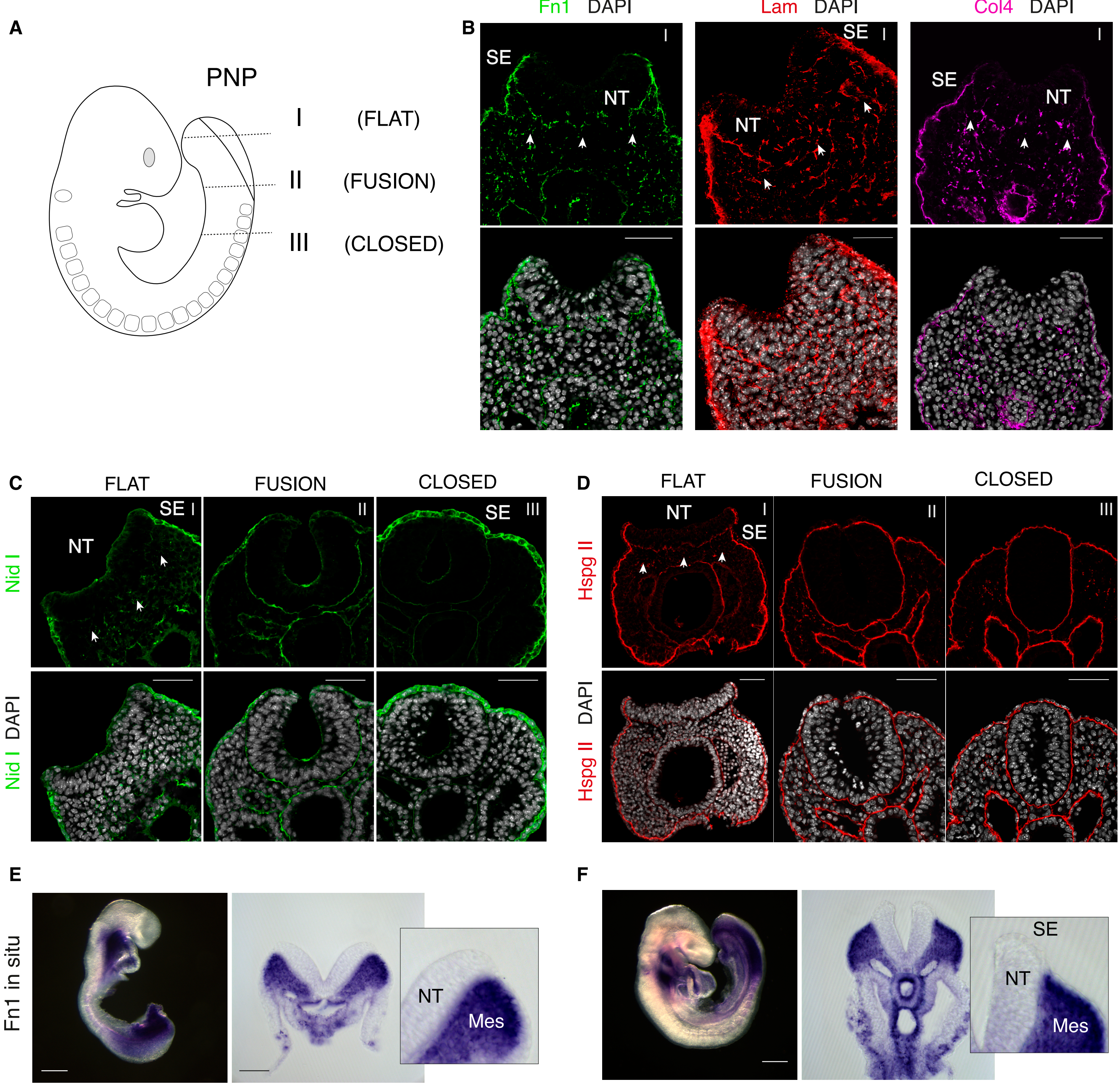
A
PNP
I (FLAT)
II (FUSION)
III (CLOSED)
B
Fn1 DAPI
Lam DAPI
Col4 DAPI
SE
NT
I
C
FLAT
FUSION
CLOSED
Nid I
Nid I DAPI
II
III
D
Hspg II
Hspg II DAPI
E
Fn1 in situ
Mes
F

Figure S2

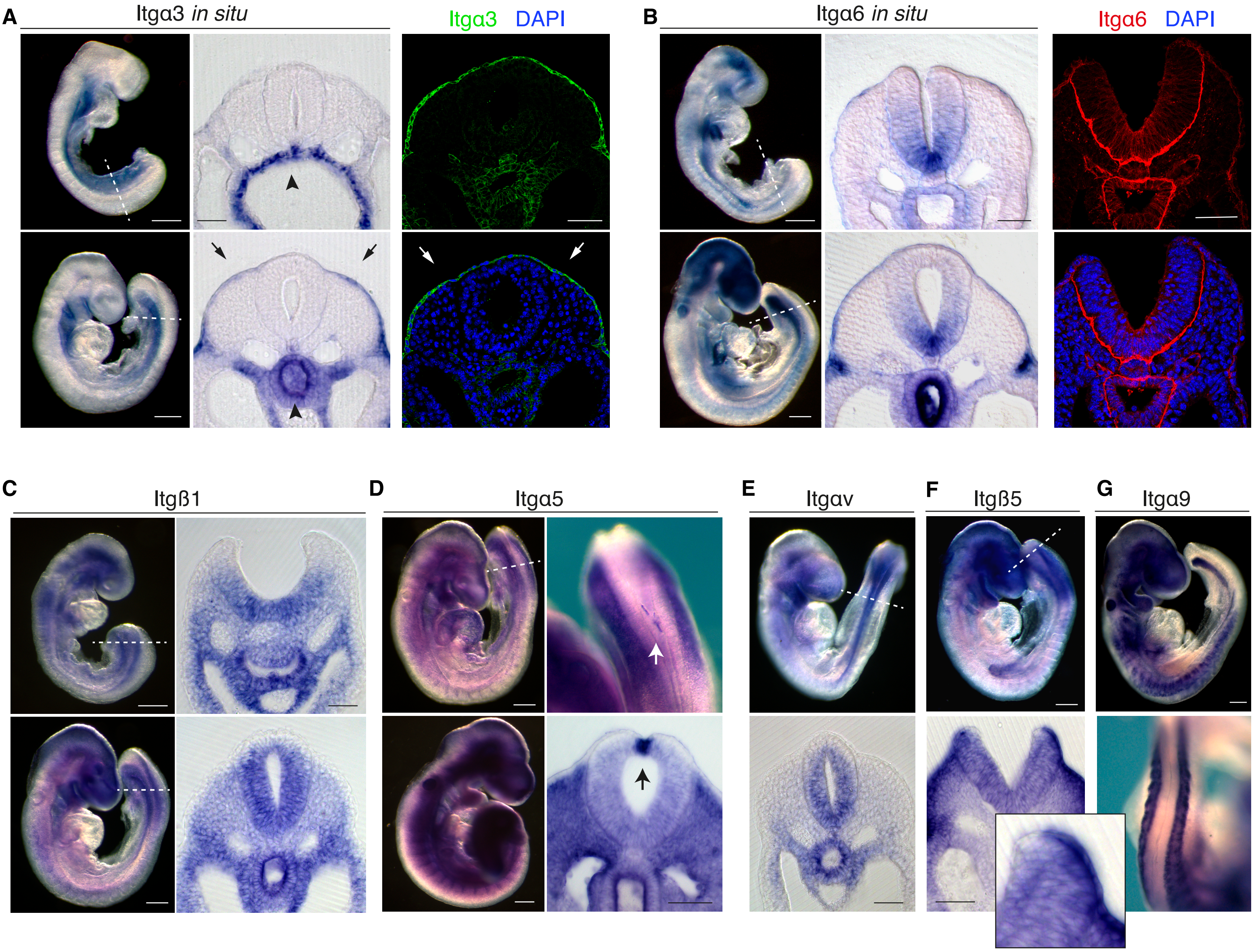
A
Itgα3 *in situ*
Itgα3 DAPI
B
Itgα6 *in situ*
Itgα6 DAPI
C
Itgβ1
D
Itgα5
E
Itgav
F
Itgβ5
G
Itgα9

Figure S3

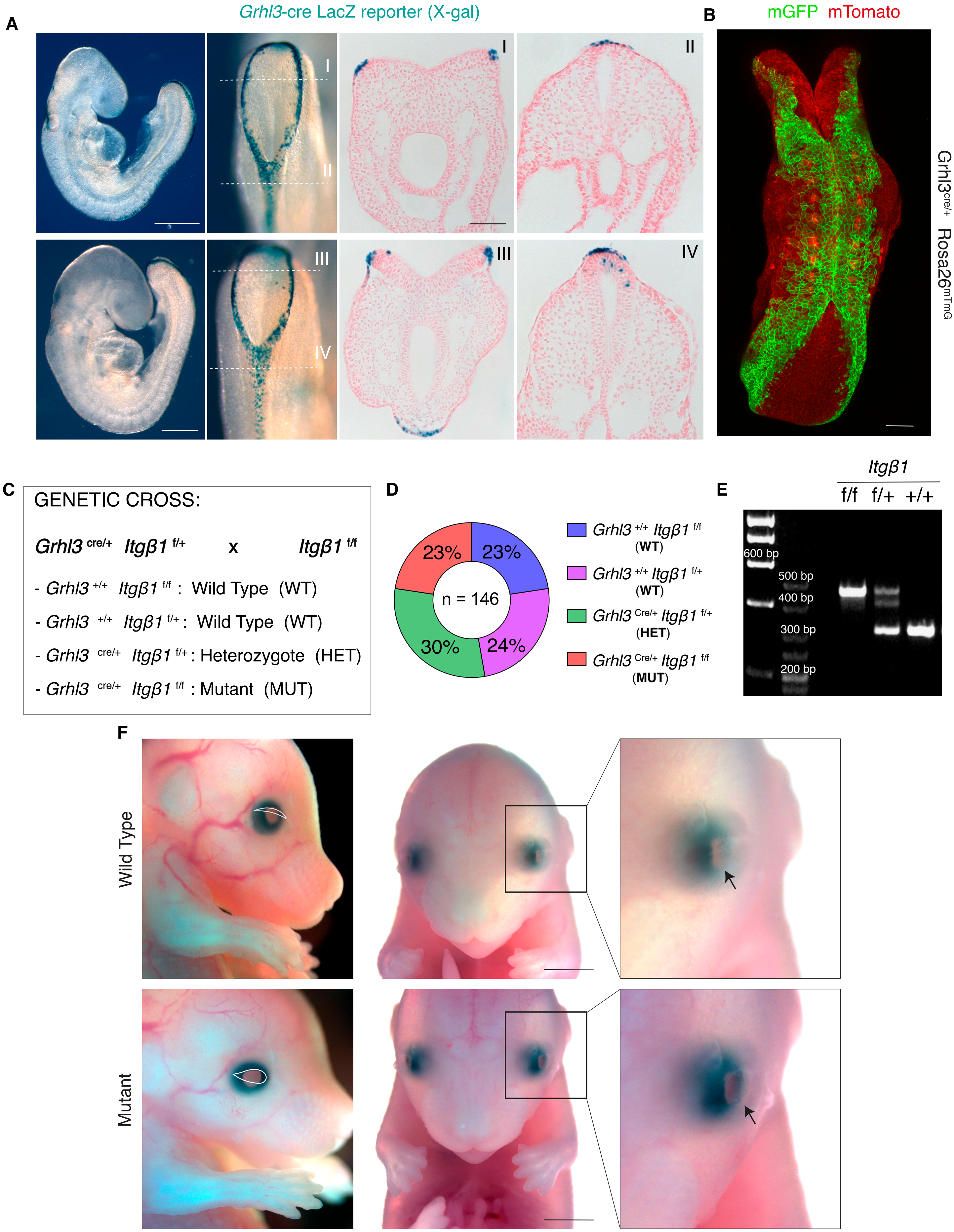
A
Grhl3-cre LacZ reporter (X-gal)
I
II
III
IV
B
mGFP mTomato
Grhl3cre/+ Rosa26mTmG
C
GENETIC CROSS:
Grhl3 cre/+ Itgβ1 f/+ x Itgβ1 f/f
- Grhl3 +/+ Itgβ1 f/f : Wild Type (WT)
- Grhl3 +/+ Itgβ1 f/+ : Wild Type (WT)
- Grhl3 cre/+ Itgβ1 f/+ : Heterozygote (HET)
- Grhl3 cre/+ Itgβ1 f/f : Mutant (MUT)
D
23%
23%
n = 146
30%
24%
Grhl3 +/+ Itgβ1 f/f (WT)
Grhl3 +/+ Itgβ1 f/+ (WT)
Grhl3 Cre/+ Itgβ1 f/+ (HET)
Grhl3 Cre/+ Itgβ1 f/f (MUT)
E
Itgβ1
f/f f/+ +/+
600 bp
500 bp
400 bp
300 bp
200 bp
F
Wild Type
Mutant

Figure S4

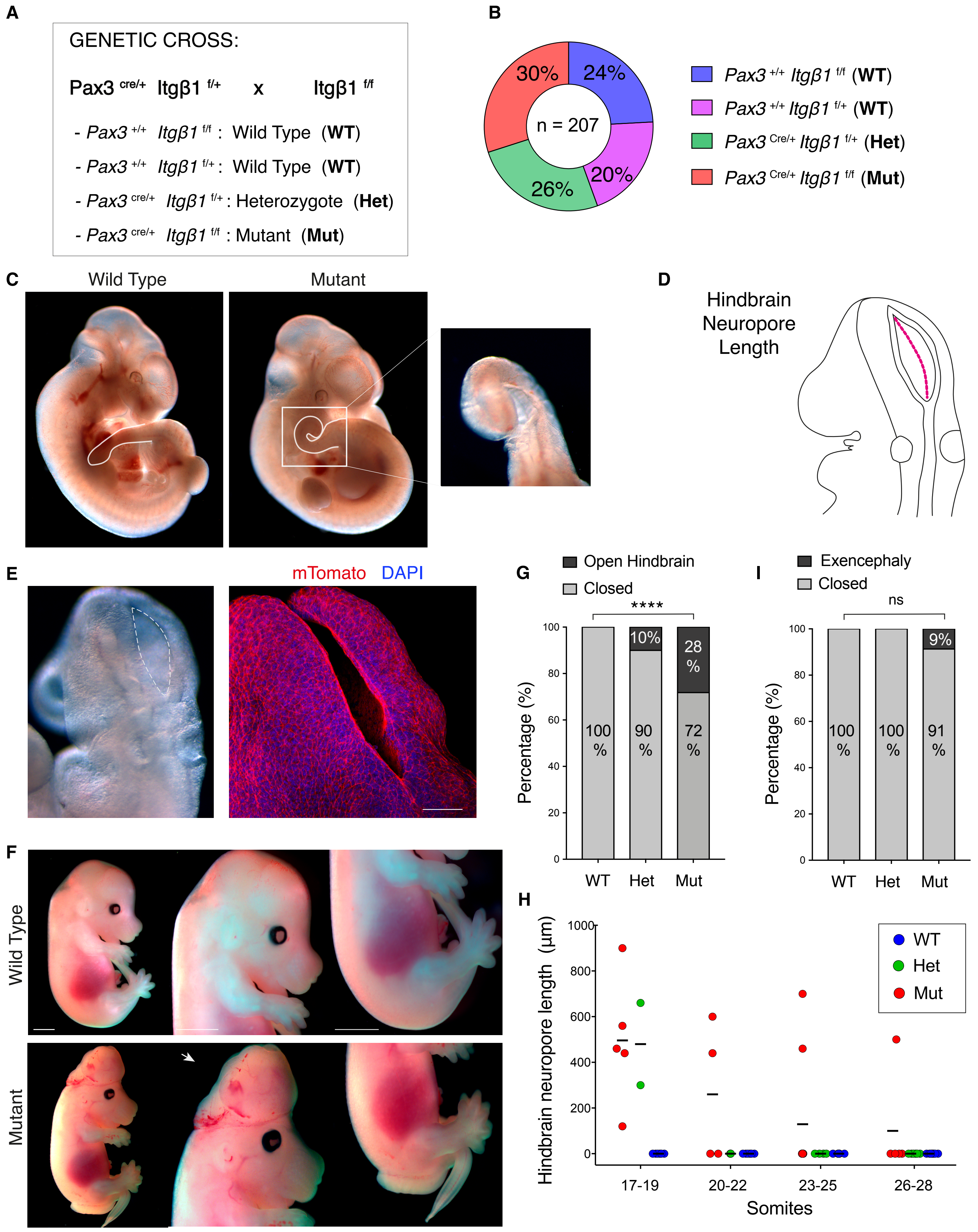
A
GENETIC CROSS:
Pax3 cre/+ Itgβ1 f/+ x Itgβ1 f/f
- Pax3 +/+ Itgβ1 f/f : Wild Type (WT)
- Pax3 +/+ Itgβ1 f/+ : Wild Type (WT)
- Pax3 cre/+ Itgβ1 f/+ : Heterozygote (Het)
- Pax3 cre/+ Itgβ1 f/f : Mutant (Mut)
B
n = 207
30%
24%
20%
26%
Pax3 +/+ Itgβ1 f/f (WT)
Pax3 +/+ Itgβ1 f/+ (WT)
Pax3 Cre/+ Itgβ1 f/+ (Het)
Pax3 Cre/+ Itgβ1 f/f (Mut)
C
Wild Type
Mutant
D
Hindbrain Neuropore Length
E
mTomato DAPI
F
Wild Type
Mutant
G
Open Hindbrain
Closed
****
Percentage (%)
100 %
10%
90 %
28 %
72 %
WT
Het
Mut
I
Exencephaly
Closed
ns
Percentage (%)
100 %
100 %
9%
91 %
WT
Het
Mut
H
Hindbrain neuropore length (µm)
WT
Het
Mut
17-19
20-22
23-25
26-28
Somites

Figure S5

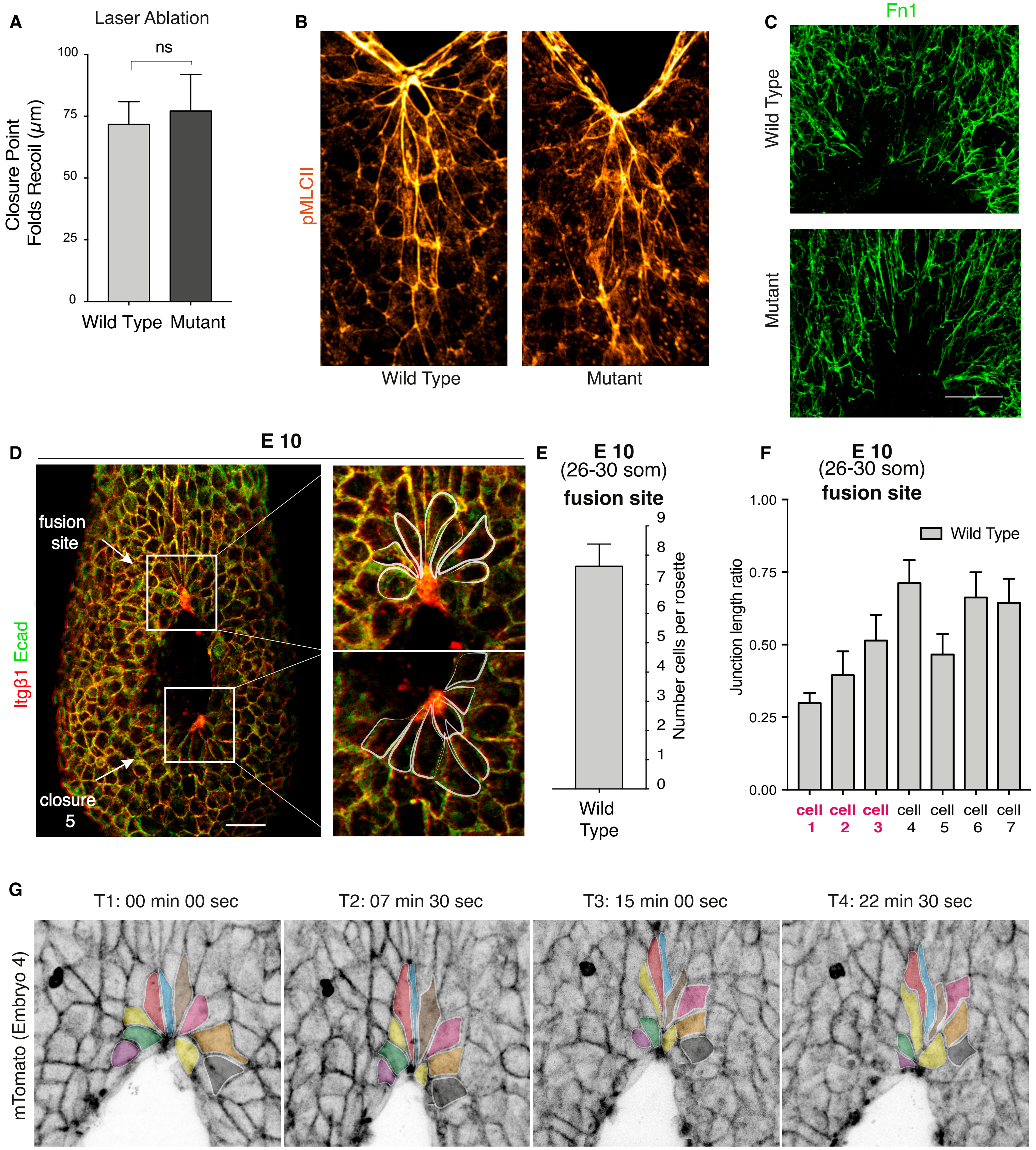

A
Laser Ablation
ns
Closure Point Folds Recoil (μm)
100
75
50
25
0
Wild Type
Mutant
B
pMLCII
Wild Type
Mutant
C
Fn1
Wild Type
Mutant
D
E 10
fusion site
closure 5
Itgβ1 Ecad
E
E 10
(26-30 som)
fusion site
Number cells per rosette
9
8
7
6
5
4
3
2
1
0
Wild Type
F
E 10
(26-30 som)
fusion site
Wild Type
Junction length ratio
1.00
0.75
0.50
0.25
0.00
cell 1
cell 2
cell 3
cell 4
cell 5
cell 6
cell 7
G
T1: 00 min 00 sec
T2: 07 min 30 sec
T3: 15 min 00 sec
T4: 22 min 30 sec
mTomato (Embryo 4)

SUPPLEMENTAL FIGURES

Figure S1. Related to Figure 1. Temporal and Spatial Heterogeneity of Basement Membranes during Neural Tube Closure. (**A**) Axial level of transverse embryonic sections: (I) 'flat' NE in the caudal PNP; (II) elevated neural folds with onset of 'fusion'; (III) recently 'closed' neural tube. (**B**) Immunofluorescence for fibronectin (Fn1), laminin (Lam) and collagen type 4 (Col4) in the most caudal PNP (I). BM deposition beneath the neuroepithelium (NT) has initiated but is fragmentary and incomplete (arrows), in contrast to the continuous BM underlying the surface ectoderm (SE) (Stages: 25 som (Fn1); 12 som (Lam); 20 som (Col4)). (**C**, **D**) Nidogen-I (Nid 1) (C) and Perlecan (Hspg II) (D) are present in the nascent BM beneath the flat NE (I, arrows), and give rise to a continuous mature BM upon elevation of the neural folds (II). Nidogen-1 expression is distributed peri-cellularly in the SE. (Stages: 21 som (C), 24 som (D)). (**E, F**) *In situ* hybridisation analysis of *Fn1*: transcripts are confined to paraxial mesoderm (Mes) and are not detectable in NE nor SE (Stages: 7 som (E); 17 som (F)). Scale bars: 50 μm (B-D); 200 μm (E-F, whole mount); 100 μm (E-F, sections).

Figure S2. Related to Figure 2. Gene Expression and Localisation of Integrins. *In situ* hybridisation (whole mount, left; sections, middle) and immunofluorescence (right) for each integrin subunit. Dotted lines on whole mounts indicate levels of sections. (**A**) *Itgα3* is transcribed in SE from 16 somite stage (arrows), and also in gut endoderm (arrowheads). Stage: 9 som (top), 16 som (bottom). Itgα3 protein shows a peri-cellular distribution around SE cells. Stage: 16 som. (**B**) *Itgα6* is transcribed in ventral neural tube (future floor plate) and SE. Stage: 9 som (top), 25 som (bottom). Itgα6 protein is intensely expressed at the basal neuroepithelial surface, with a ventro-dorsal gradient. Stage: 24 som. (**C**) *Itgβ1* is widely expressed in all tissues. Stage: 10 som (top), 19 som (bottom). See Figure 3C,D for protein expression. (**D**) *Itgα5* is transcribed in mesoderm and also at the site of neural fold fusion, as detected in whole mounts (white arrow) and sections (black arrow). Note intense expression of the Itgα5 subunit can be detected at the neural fold tips just before they come into contact, and soon after closure at the zippering point. Stage: 20 som (top), 26 som (bottom). (**E**) *Itgαv* is expressed in NE and gut endoderm. Stage: 19 som. (**F**) *Itgβ5* is transcribed in NE, mesoderm and most intensely in SE. The neural fold tips are devoid of *Itgβ5* (zoom view). Stage: 23 som. (**G**) *Itgα9* is exclusively expressed in the paraxial mesoderm. Stage: 25 som. Scale bars: 200 μm (whole mounts), 50 μm (sections).

Figure S3. Related to Figure 3. Genetic Ablation of Integrin β1 in the Surface Ectoderm by *Grhl3*-Cre. (**A**) *Grhl3*-Cre expression as detected by the endogenous LacZ cassette inserted within the *Grhl3* locus

(nuclear staining). At E9.0 (top), Cre is expressed exclusively in the dorsal SE. At E9.5 (bottom), in addition to SE, a few dorsal neuroepithelial cells are Cre-positive. Stage: 13 som (top) and 21 som (bottom). (**B**) Lineage tracing using the reporter Rosa26^{mTmG} shows *Grhl3*-Cre mediated excision (mGFP expression) in the SE of a whole-mount E9.0 embryo, whereas non-recombined cells express mTomato. Stage: 8 som. (**C**) Genetic cross used in generation of embryos lacking integrin-*β1* in the *Grhl3* expression domain. (**D**) Frequency of the four genotypes amongst all embryos (E9.5-15.5) does not differ from Mendelian expectations (Chi-square test: $p>0.05$; n = 146). (**E**) PCR genotyping of the *Itgβ1* floxed allele. (**F**) At E15.5, mutant foetuses (2/2) fail to complete eyelid closure, compared with Het (0/5) or WT (0/5), which exhibit fully closed eyelids. Scale bars: 200 μm (A, whole mount); 100 μm (A, sections, B), 2 mm (F)

Figure S4. Related to Figure 4. Genetic Ablation of Integrin β1 in the Dorsal NE by *Pax3*-Cre. (**A**) Genetic cross used in generation of embryos lacking integrin-*β1* in the *Pax3* expression domain. (**B**) Frequency of the four genotypes amongst all embryos (E9.5-14.5) does not differ from Mendelian expectation (Chi-square test: $p>0.05$; n = 207). (**C**) At E11, a proportion of Mut embryos display a small spina bifida, together with tail flexion defect, whereas WT embryos exhibit normal spine and tail development. Stage: 42 som. (**D**) Hindbrain neuropore length was measured as shown by dotted line. (**E**, **G**) At E9.5, 28% of mutant (Mut) embryos show delayed closure of the hindbrain neuropore (E), compared to WT (G; Fisher's exact test: $p<0.0001$; n-values: 52, WT; 30, Het; 39, Mut). (**H**) WT and heterozygous (Het) embryos have closed hindbrains beyond 17 and 20 somites, respectively, whereas some Mut embryos display open hindbrain at later stages. (**F**, **I**) At E14.5, 9% of mutants fail in hindbrain closure and exhibit exencephaly (F, arrow), non-significant compared with WT (I; Fisher's exact test; n-values: 40, WT; 23, Het; 23, Mut). Scale bars: 500 μm (A); 100 μm (I); 2 mm (E).

Figure S5. Related to Figure 6-7. Biomechanical, Morphometric and Live Imaging Analysis.
(**A**) Laser ablation shows identical recoil at site of closure (Mann-Whitney, $p>0.05$, no=9 WT; no=7 Mut). Stages: 20-24 som. (**B**) Analysis of phosphorylated myosin along the dorsal region of the recently closed neural tube shows no detectable change in active contractility near the site of fusion between wild type and mutant embryos. (**C**) Fibronectin fibrils in the dorsal BM orient radially towards the site of fusion and are similarly present in both WT and Mut embryos (n=11 embryos analysed). (**D**) Late in spinal closure at E10.0 (26-30 som), semi-rosette arrangements of SE cells can be visualised both at the site of rostral-to-caudal zippering and at Closure 5. Note focal expression of integrin β1 at the vertices of both semi-rosette structures. (**E**) The semi-rosette-like structure at the main site of zippering at E10.0 (26-30 som) contains on average 7 cells (number embryos: n=8 WT; Mut N/A). (**F**)

Analysis of SE junction length ratio at E10.0 (26-30 som) at the site of zippering. SE cells near the site of fusion (cells 1-3) display a progressive trend towards proximal junction shortening (wedge-shaped morphology) in contrast to cells bordering the open PNP which have a more rectangular morphology with a junction length ratio of > 0.5 (number embryos: n=8 WT (112 cells)). (**G**) Additional example of live imaging at E9.5 (embryo 4) showing proximal junction remodelling and rosette dynamics as in Figure 7H. Scale bars: 50 μm (C-D).
